# Cryptosporidiosis Modulates the Gut Microbiome and Metabolism in a Murine Infection Model

**DOI:** 10.3390/metabo11060380

**Published:** 2021-06-11

**Authors:** Avinash V. Karpe, Melanie L. Hutton, Steven J. Mileto, Meagan L. James, Chris Evans, Rohan M. Shah, Amol B. Ghodke, Katie E. Hillyer, Suzanne S. Metcalfe, Jian-Wei Liu, Tom Walsh, Dena Lyras, Enzo A. Palombo, David J. Beale

**Affiliations:** 1 Land and Water, Commonwealth Scientific and Industrial Research Organization, Ecosciences Precinct, Dutton Park, QLD 4102, Australia; avinash.karpe@csiro.au (A.V.K.); r.shah@csiro.au (R.M.S.); katie.hillyer@csiro.au (K.E.H.); suzanne.metcalfe@csiro.au (S.S.M.); 2 Infection and Immunity Program, Monash Biomedicine Discovery Institute and Department of Microbiology, Monash University, Clayton, VIC 3800, Australia; melanie.hutton@monash.edu (M.L.H.); steven.mileto@monash.edu (S.J.M.); meagan.james@monash.edu (M.L.J.); chris.evans@monash.edu (C.E.); dena.lyras@monash.edu (D.L.); 3 Department of Chemistry and Biotechnology, Swinburne University of Technology, Hawthorn, VIC 3122, Australia; epalombo@swin.edu.au; 4 Queensland Alliance for Agriculture and Food Innovation, Department of Horticulture, The University of Queensland, St Lucia, QLD 4072, Australia; a.ghodke@uq.edu.au; 5 BIO21 Institute, School of Biosciences, The University of Melbourne, Parkville, VIC 3010, Australia; 6 Land and Water, Commonwealth Scientific and Industrial Research Organization Research and Innovation Park, Acton, ACT 2601, Australia; jian-wei.liu@csiro.au (J.-W.L.); tom.walsh@csiro.au (T.W.)

**Keywords:** interactomics, host–parasite–microbiome relationships, extra-intestinal effects, D-amino acid/SCFA-induced modulation, yeast ubiquinone salvation

## Abstract

Cryptosporidiosis is a major human health concern globally. Despite well-established methods, misdiagnosis remains common. Our understanding of the cryptosporidiosis biochemical mechanism remains limited, compounding the difficulty of clinical diagnosis. Here, we used a systems biology approach to investigate the underlying biochemical interactions in C57BL/6J mice infected with *Cryptosporidium parvum*. Faecal samples were collected daily following infection. Blood, liver tissues and luminal contents were collected 10 days post infection. High-resolution liquid chromatography and low-resolution gas chromatography coupled with mass spectrometry were used to analyse the proteomes and metabolomes of these samples. Faeces and luminal contents were additionally subjected to 16S rRNA gene sequencing. Univariate and multivariate statistical analysis of the acquired data illustrated altered host and microbial energy pathways during infection. Glycolysis/citrate cycle metabolites were depleted, while short-chain fatty acids and D-amino acids accumulated. An increased abundance of bacteria associated with a stressed gut environment was seen. Host proteins involved in energy pathways and *Lactobacillus* glyceraldehyde-3-phosphate dehydrogenase were upregulated during cryptosporidiosis. Liver oxalate also increased during infection. Microbiome–parasite relationships were observed to be more influential than the host–parasite association in mediating major biochemical changes in the mouse gut during cryptosporidiosis. Defining this parasite–microbiome interaction is the first step towards building a comprehensive cryptosporidiosis model towards biomarker discovery, and rapid and accurate diagnostics.

## 1. Introduction

Enteric protozoal infections are a major human health concern globally, causing malnutrition through the loss of appetite, decreased nutrient absorption, and increased catabolism of nutrient reserves due to inflammation and diarrhoea [1]. In particular, cryptosporidiosis is a global endemic infection causing about 4.2 million annual reported cases and >7.5 million disability-adjusted life-years, with a high impact on children aged 4 years or below and immunocompromised individuals [2]. The infection is caused by members of *Cryptosporidium* spp., such as *C. parvum* and *C*. *hominis* among others (henceforth indicated as *Cryptosporidium*). *Cryptosporidium* are highly specialised obligate apicomplexan parasites that transmit via the faecal–oral route from sources such as drinking water, or recreational waters contaminated with raw sewage and/or animal faeces. Due to their ability to infect humans and other mammals, they are considered to be ubiquitous parasites; some species are anthroponotic (*C. hominis*), while others are zoonotic (*C. parvum*) [3].

*Cryptosporidium* infections are limited to the epithelial lining of the gastrointestinal tract, causing minimal invasion and penetration through mucosal layers, and are known to be autophagic [4]. *Cryptosporidium* lacks numerous metabolic systems and must interact with its host to compensate for these deficiencies [5,6]. This significant intertwined relationship between a host and *Cryptosporidium* is, therefore, highly complex but is only partially understood. It has been shown that cryptosporidiosis causes long-term pan-body effects such as weight loss, abdominal, eye, and joint pain, and, in some cases, irritable bowel syndrome (IBS) [7]. However, the molecular and biochemical mechanisms that result in these broad effects are not well understood. One of the primary reasons for this is because most of the biochemical profiling of cryptosporidiosis, in mouse models or clinical trials, is conducted principally through analysis of faeces [7,8], and, in some limited examples, caecal samples [9]. However, pathology and epidemiology data indicate the small intestine sections of jejunum and ileum to be main sites of *Cryptosporidium* colonisation and replication [10]. Therefore, knowing the molecular and biochemical interactions within these regions will provide a better understanding of the host–parasite–microbiome interactions and any downstream effects of these activities in and beyond the gut.

In this context, a multi-omics approach has the potential to provide broader systems biology information relating to cryptosporidiosis. Omics platforms such as genomics, proteomics, and metabolomics, alone or in combination, have provided new insights that have been valuable in preventative health [11,12], toxicology, and medicine [13,14]. The high sensitivities and specificities of multi-omics platforms provide excellent discrimination between samples and treatment types, and have been applied to study environmental, clinical, and natural medicine systems [15,16,17]. Metabolomics and genomics have been independently applied to understand the *Cryptosporidium* life cycle in aquatic systems [18,19], and in-vitro studies [4,6]. Metabolomics or gut microbial community genomics studies have also been performed separately on the infected host [8,20].

In this study, we used multi-omics platforms to investigate biochemical interactions between *C. parvum* and a murine host as the parasite passes through various gut sections, and how the effects of these interactions extend beyond the gut. To define the host-specific interactions of *C. parvum*, infection with the bacterium uropathogenic *Escherichia coli* (UPEC) [21] and the eukaryotic pathogen *Giardia lamblia* were used for comparative purposes. Gut infection with UPEC does not appear to cause disease symptoms in humans or mice [22], while giardiasis results in similar symptoms to those seen in cryptosporidiosis [3]. Untargeted metabolomics, proteomics and microbiome 16S rRNA gene sequencing were applied to numerous body tissues and gut washes after infection with the abovementioned infectious agents. We examined how, during cryptosporidiosis in mice, the enteric microbial community profile is altered and which metabolic processes change throughout the mouse intestinal tract and in extra-intestinal tissues.

## 2. Results

### 2.1. Mouse Strain Selection for Multi-Omics Studies

**Figure 1:**
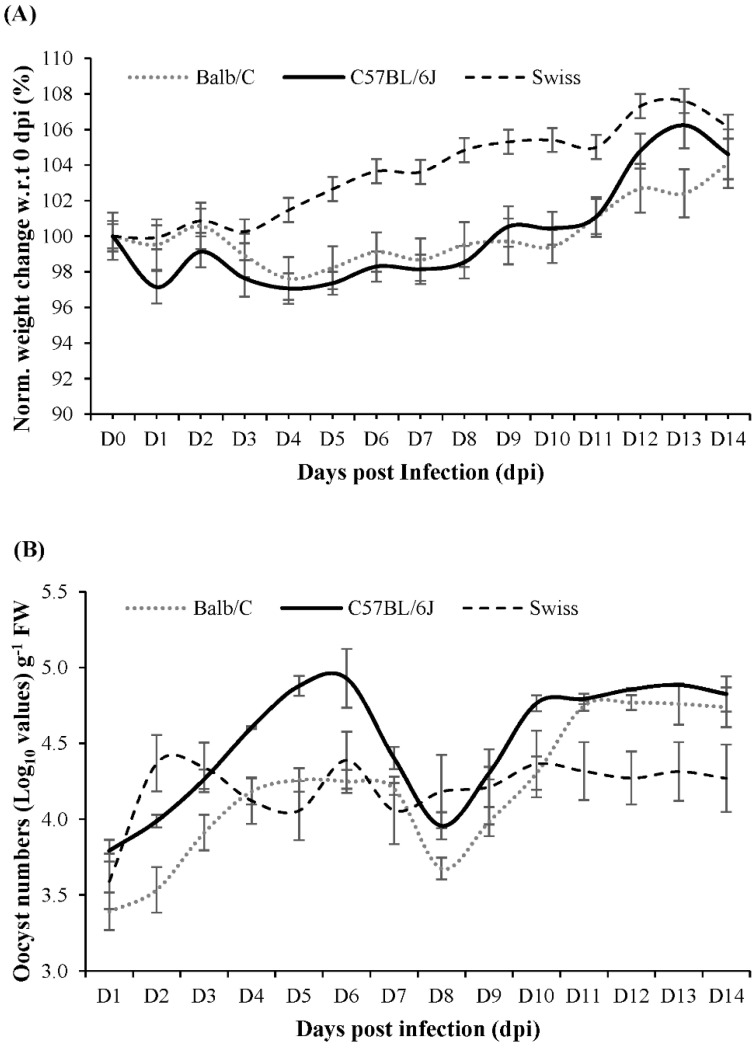
(**A**) Daily percentage weight change (normalized with respect to 0 dpi) in Balb/C, Swiss and C57BL6/J mice that were infected with *C. parvum* and monitored for 14 dpi (**B**) *C. parvum* count (Log_10_ growth in terms of oocyst count), per gram fresh weight of faeces. The error bars indicate standard deviation (*n* = 3, *p*-value ≤ 0.05).

The C57BL/6J strain was selected for this study, based on the results of a 14-day pilot study where *C. parvum* infection was compared in Balb/C, C57BL/6J and Swiss mice. During the pilot study, none of the infected mouse strains showed diarrhoeal symptoms. However, C57BL/6J mice had a slightly higher relative weight loss until 6 days post infection (dpi) before steadily gaining weight (Figure 1). In addition, C57BL/6J mice showed a greater oocyst release in their faeces compared to Balb/C and Swiss mice. Based on the pilot study results, sampling for the follow-up main study was conducted for a duration of 0–10 dpi.

### 2.2. Gut Metabolism and Major Metabolic Pathways

The main study analysed biochemical interactions in the infected host’s gastrointestinal tract during cryptosporidiosis, which were assessed by (i) the response of the host’s system and gut microbiome to the parasite in the individual gut sections, and (ii) the effects on non-gut organs in terms of altered protein and metabolite profiles.

The genomic analysis of luminal contents and faeces, performed via diversity metric indices such as Good’s coverage, indicated good data quality and sequencing depth towards representational operational taxonomic units (OTUs) (Table S1A). The sequencing analysis indicated an average of 24,239 ± 51 feature counts in both uninfected and *Cryptosporidium*-infected groups.

Proteomic analysis indicated 4239 host expressed proteins, with a good fit (R^2^X = 0.88, R^2^Y = 0.799) but average predictability (Q^2^ = 0.41) (Figure S2A,B) [14] of the proteomic model during the infection. Searches against 43 microbial UniProt databases showed the number of expressed microbiome proteins in the gut to be 30 (duodenum), 63 (jejunum), 142 (ileum), 874 (caecum), 815 (colon) and 956 (faeces).

**Figure 2:**
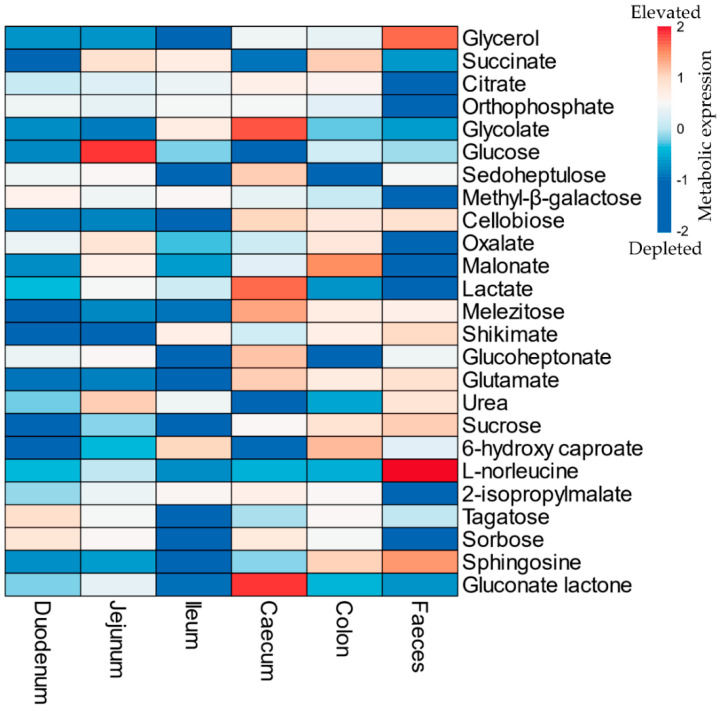
List of top 25 metabolites (in descending order) with significantly high variable importance in projection (VIP) scores in the mouse gut during *Cryptosporidium* infection. The colors refer to relative depletion (blue) and elevation (red) of the metabolites in the gut of infected mice with respect to the uninfected mice (Refer to Table S13 for data). Note: The scale indicates log transformed and pareto scaled values for the elevated (red) or depleted (blue) metabolites during the infection.

The metabolome output showed the presence of 162 identified metabolites across all the analysed samples with generally good fit (R^2^ > 0.5) and predictability (Q^2^ > 0.8) (Table S2, Figure S3). During cryptosporidiosis, most metabolites depleted (FC < 0.5) in the small intestine. On the contrary, the number of elevated metabolites increased in the caecum and colon during infection (Figure 2, Tables S3–S8).

The integrated joint-pathway analysis of metabolic-proteomic datasets showed 69 key metabolic pathways being expressed, of which 10 were statistically significant with respect to uninfected mice (Holm adjusted *p*-value ≤ 0.05) (Table 1).

### 2.3. Gut Microbiome Response and Altered Energy Metabolic Pathways during Cryptosporidiosis

**Figure 3:**
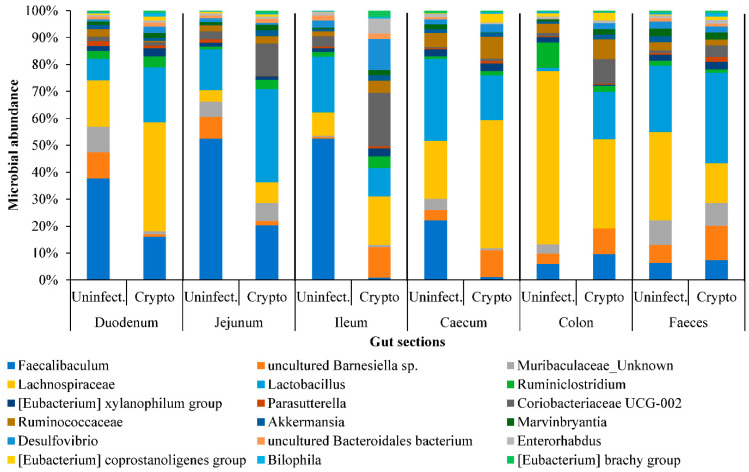
The abundance of predominant bacterial genera across regions of the intestinal system of uninfected and *Cryptosporidium*-infected mice. Individual contribution is presented in Table S1B.

The 16S rRNA gene analysis identified 71 bacterial genera. Of these, 22 genera were represented in all luminal contents (Figure 3). Whilst *Faecalibaculum*, *Barnesiella*, and *Lactobacillus* were abundant in the small intestine, the *Ruminococcaceae* population increased in the caecum and colon (Figure 3). It was also observed that during cryptosporidiosis, *Faecalibaculum* and *Lachnospiraceae* showed gradual depletion from duodenum onwards, while *Lactobacillus*, *Lachnospiraceae*, *Desulphovibrio*, and *Coriobacteria* populations increased, especially in the jejunum and ileum. It was determined that by 10 dpi, populations of *Coriobacteriaceae*, *Ruminococcaceae* and *Lachnospiraceae* species in the faeces increased considerably (Figure S1C), with the exception of *Lactobacillus*.

**Figure 4:**
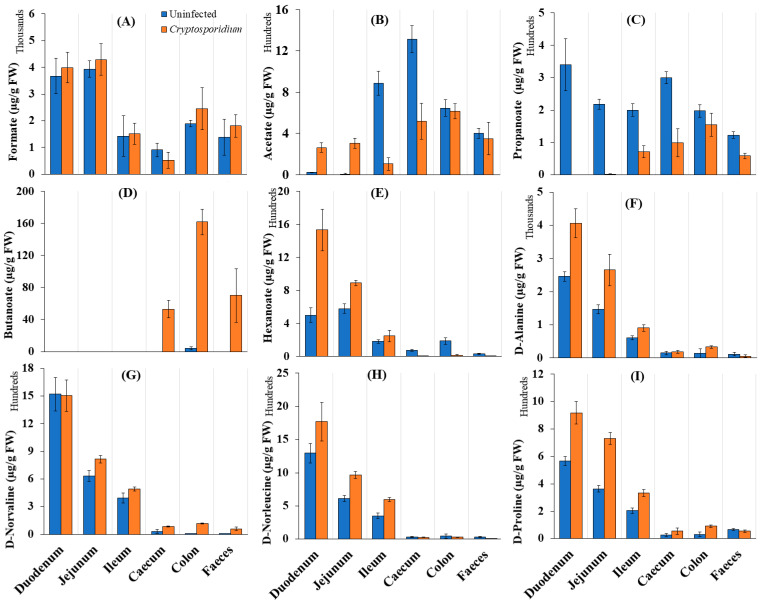
Distribution of (**A**–**E**) short chain fatty acids (SCFAs) and (**F**–**I**) D-amino acids across various regions of the intestinal tract (µg/g FW of samples) of uninfected and *Cryptosporidium*-infected mice. Note: The error bars represent standard deviation between the experimental replicates of each organ (*n* = 5).

We examined whether cryptosporidiosis-induced changes in the abovementioned microbiota composition indeed affected SCFA production in the mouse gut. Among SCFAs, butanoate showed a considerable increase in *Cryptosporidium*-infected mice. Butanoate levels particularly increased in the caecum and colon during cryptosporidiosis (Figure 4A–E). Interestingly, a higher accumulation of D-amino acids, especially D-alanine, D-norleucine and D-proline, was seen in the small intestine (Figure 4F–I), where an increased abundance of *Lactobacillus*, *Lachnoclostridium*, *Coriobacteriaceae* and *Lachnospiraceae* (Figure S1C,D) was seen.

**Figure 5:**
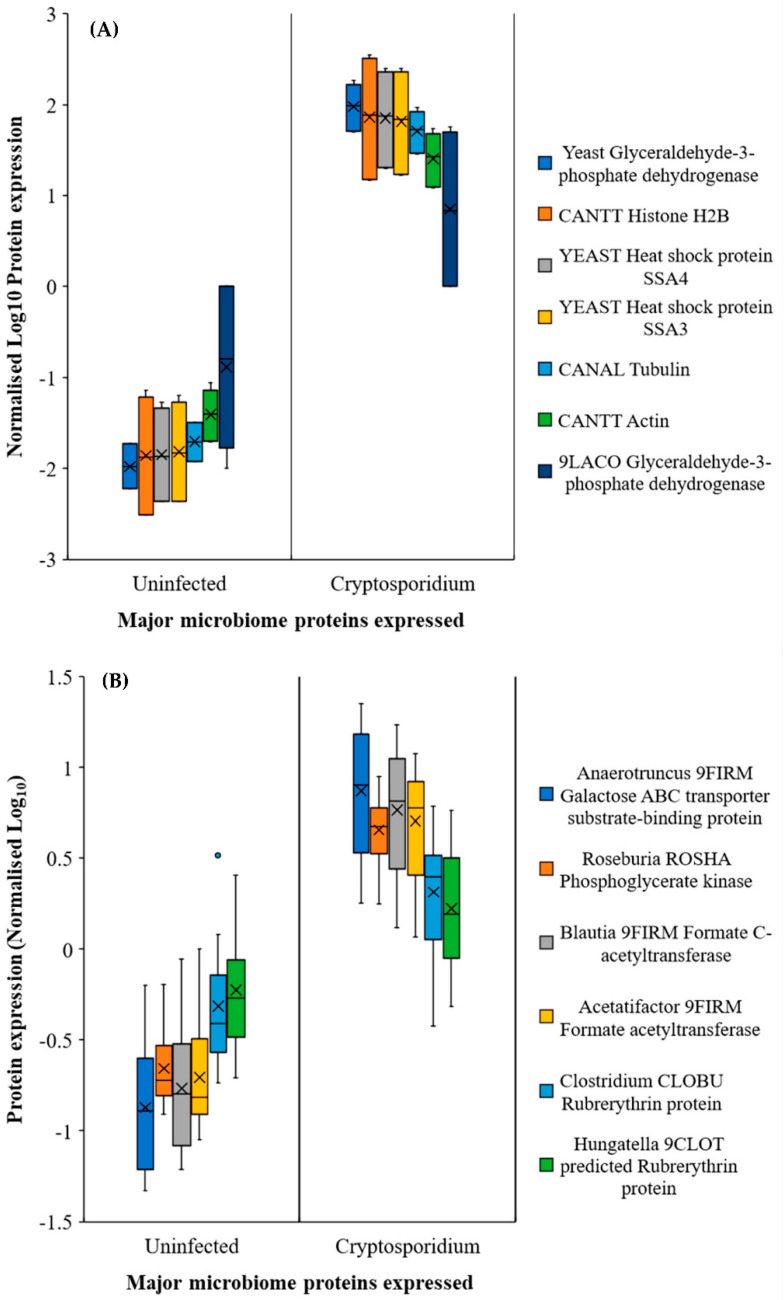
Major proteins expressed by microbial community in response to cryptosporidiosis in (**A**) the jejunum–ileum and (**B**) the caecum–colon region. Note: Error bars represent standard deviation between the experimental replicates (*n* = 10 for **A** and **B**).

We determined that an increase in *Lactobacillus* (or similar bacterial) population (Figure 3 and Figure S1D) was also indicated by the increased expression of glycolysis and fatty acid metabolism related proteins (Figure 5A) in the small intestine. In addition, one of the surprising, unforeseen observations was a considerable increase in yeast protein expression in the jejunum and ileum during the infection (Note: all Uniprot IDs and Individual database IDs are provided in Supplementary Materials section). These proteins included histone H4 proteins of *Candida* (C5M3N6, FC = 1.15) and *Saccharomyces* (P02309, FC = 1.15), showing similar upregulated expression to *Cryptosporidium* histone H4 proteins (Q5CV68, FC = 1.15). Additionally, significantly upregulated glycolysis pathway proteins such as glyceraldehyde-3-phosphate dehydrogenases from yeasts (P00360, cFC = 11.02) and *Lactobacillus* (A0A062X383, cFC = 1.41) were seen in the jejunum–ileum section during cryptosporidiosis (Figure 5A, Supporting Dataset 1). In the ileum, *Saccharomyces* mitochondrial dihydrolipoyl dehydrogenase (P09624; FC = 83.08) and *Candida* malate dehydrogenases (C5M2D7, FC = 817.11 and Q5AMP4, FC = 547.99) were highly upregulated.

In the caecum and colon, the predominantly expressed microbial proteins were mainly related to the glycolysis pathway, leading to fatty acid synthesis and oxidative stress protection (e.g., rubrerythrins) (Figure 5B). The proteomic expressions confirmed the functional outputs of the gut microbiome population. Besides the major species (Figure 4), bacteria that were either localised to a few sections or had low population numbers showed considerably high metabolic activities, especially under the increasingly anaerobic environments of the gut. For example, *Blautia*, a low population species, was elevated in the caecum and colon of infected mice with respect to the uninfected mice (Figure 5B). It was also worth noting that although the *Blautia* populations were low throughout the intestine (<0.5% of the total prokaryotic microbiome), they showed considerable metabolic activity, reflected through the increased expression of *Blautia* acetyltransferase.

### 2.4. Host Response in the Gut during Cryptosporidiosis

**Figure 6:**
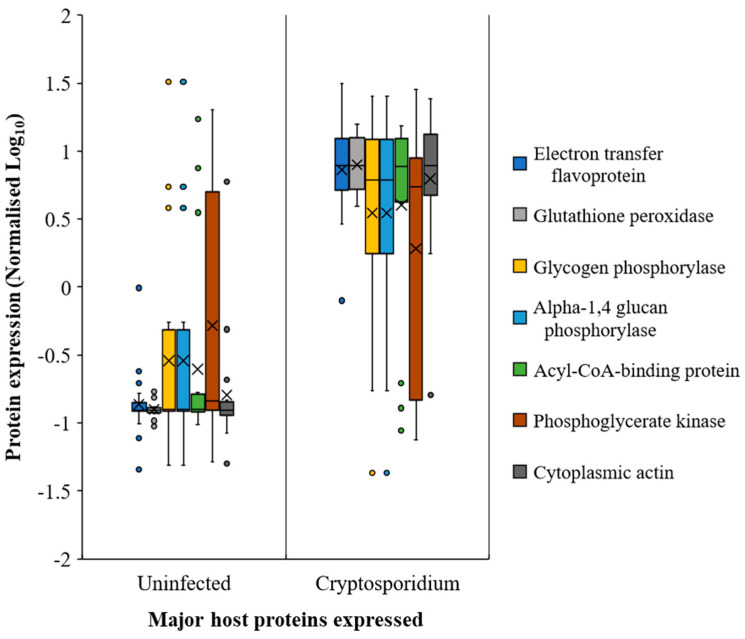
Most prominent host proteins expressed across the mouse intestine (both small and large intestine sections) upon *Cryptosporidium* infection. Note: Error bars represent standard deviation between the experimental replicates (*n* = 25).

It appeared that host protein expression increased considerably from the jejunum onwards during infection. Among the host response proteins, actins showed the highest upregulation (Figure 6 and Table S9). The glycolysis/gluconeogenesis-associated enzymes, such as glutathione peroxidase, electron transfer flavoproteins, sugar phosphorylases, and phosphoglycerate kinase, among others, also showed increased expression (Figure 6, Table S9, Supporting Dataset 2) during cryptosporidiosis, even with respect to giardiasis and UPEC infection. Certain *Cryptosporidium* proteins, although expressed throughout the small intestine, showed higher expression in ileum. These include actin (FC = 3065.1), tubulin (FC = 1040.6), and heat shock proteins (FC of HSP90 = 2483; HSP 70 = 197.2), indicative of increased parasite replication in this region during infection.

**Figure 7:**
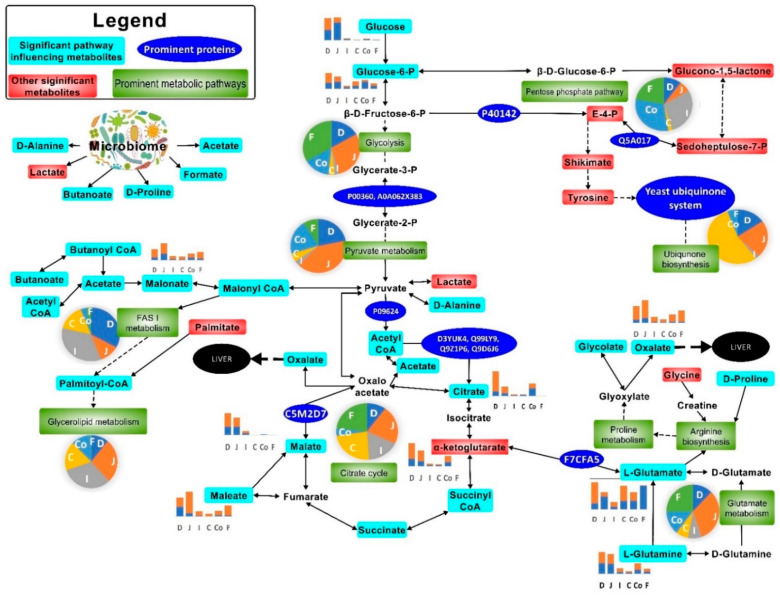
Most prominent metabolic activities in the mouse gut upon *Cryptosporidium* infection. The pie charts indicate the relative impact of pathways in the duodenum (D), jejunum (J), ileum (I), caecum (C), colon (Co), and faeces (F). The bar graphs show perturbed metabolites in uninfected (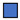) and *Cryptosporidium* (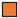) infected mice. Note: Please refer to Figure S8 for a more descriptive pathway chart.

Citrate, succinate, oxalate, malate, glycolate, and orthophosphate were catabolised more in the small intestine than the large intestine (Figure 2 and Figure 7, Tables S3–S8) during cryptosporidiosis. Proteins related to the citrate cycle and oxidative phosphorylation were expressed across the mouse intestine during cryptosporidiosis (Figure 6, Table 1). Among proteins, the highest expressions (cFC > 2) were related to oxidative phosphorylation and glycolysis (Table S9). Other energy generation pathways such as glutamate metabolism possibly assisted *Cryptosporidium* to create a proxy-citrate cycle. These involved host mitochondrial NADH dehydrogenases (ubiquinone) (Uniprot IDs: D3YUK4, Q99LY9, Q9Z1P6, and Q9D6J6; cFC = 1.35). In addition, localised glutamine synthetase upregulation (P26443, FC = 2.33) and overall glutamate dehydrogenase downregulation (F7CFA5, cFC = −0.94) indicated reduced glutamate utilisation by the host. These results indicate that considerable oxidative phosphorylation is necessary to maintain highly upregulated citrate cycle activities (Figure 7) during cryptosporidiosis [5].

To ascertain if the protein profile observed in the gut was specific to cryptosporidiosis, we compared the proteomic output during cryptosporidiosis to that obtained from a UPEC gut infection or *Giardia* infection. Actins showed similar expression across the intestine (cFC 1–6.2) for all three infections. However, proteins related to oxidative phosphorylation and glycolysis had greater expression during cryptosporidiosis when compared to UPEC infection and giardiasis (Table S9). Additionally, some proteins with elevated expression during cryptosporidiosis, such as ADP/ATP translocase (cFC = 3.59), electron transfer flavoproteins (cFC = 2.5–2.63), and acyl CoA binding proteins (cFC = 2.08), were either non-significantly (*p*-value ≥ 0.05) different or were downregulated (*p*-value ≤ 0.05) during giardiasis or UPEC infection. In addition, related proteins, such as glutathione peroxidase (cFC = 2.38) and phosphoglycerate kinase (cFC = 2.07), had significantly greater expression during cryptosporidiosis (Table S9). The analysis indicated that the proxy-citrate cycle was specifically upregulated during cryptosporidiosis compared to other gut infections (Figure 7).

### 2.5. Extra-Intestinal Effects of Cryptosporidiosis

Few studies have focused on the effects of enteric infection on non-gut organs, and, to our knowledge, no studies have addressed this for cryptosporidiosis. For this study, serum and liver were used as representative samples for measuring extra-intestinal effects, such as nutrient absorption, detoxification, and immune response.

During cryptosporidiosis, we observed downregulation of fatty acid metabolism in the serum; the major fatty acids affected were palmitoleate (FC = 0.07), oleate (0.05), and myristate (0.02) when compared to the uninfected mice (Table S10). In the liver, we observed a similar decrease, specifically of 6-hydroxy caproic acid and succinic acid (Table S11).

During cryptosporidiosis, of 1320 and 3016 expressed proteins in the serum and the liver, respectively, 327 were significantly upregulated across both (Supporting Data 2). These included haemoglobin (cFC = 10.63), myosins and selenium binding proteins, complement factors H (cFC = 3.97) and B (cFC = 3.2–3.55), immunoglobulins (cFC = 2.04–2.76), and apolipoproteins (cFC = 1.87–3.03), which showed statistically significant expression level changes when compared to the uninfected mice. Additionally, the metabolism-related proteins that are important for gluconeogenesis, the Krebs cycle, and phosphorylation, such as mitochondrial pyruvate carboxylase and creatine kinase (M-type), were highly expressed during infection (Table S12, Supporting Dataset 3).

## 3. Discussion

### 3.1. Cryptosporidiosis Dynamics in the Gut

The age of mice in the animal model is important. VanDussen et al. [9] suggested that in mice, an almost fully developed microbiome is established by the third week after birth. This was important for the current study as the microbial behavior during the infection plays a major role in the gut biochemical changes. Numerous mechanisms play a role in the *Cryptosporidium*–microbiome relationship during cryptosporidiosis that can be used to further elaborate the dynamics of this infection in humans [23]. In recent years, the role of the gut microbiome in the production of short-chain fatty acid (SCFAs) [24] has been highlighted, especially for gut disorder-induced stress during colitis or irritable bowel diseases (IBD) [17]. Synthesis and metabolism of SCFAs by the gut microbiome modulate inflammatory cytokine activity [25], especially by increased butanoate and propionate production in the caecum and colon [24]. Among the gut microbial community, *Faecalibaculum* and members of the *Erysipelotrichaceae* are known to produce high levels of lactate and SCFAs such as butanoate [26,27]. Additionally, *Blautia* and *Lachnospiraceae* contribute towards pyruvate metabolism to drive SCFA biosynthesis [28]. In the current study, increased *Blautia* and *Roseburia* populations and an upregulation of their formate C-acetyltransferases, combined with the minor elevation in the *Faecalibaculum* populations, correlated with elevated acetate and butanoate levels in the caecum and colon. We observed that bacteria such as *Coriobacteriaceae* and *Lactobacillus* increased in abundance during *Cryptosporidium* infection, especially in the small intestine. *Coriobacteriaceae* have been demonstrated to modulate glucose metabolism [29]. Similarly, the increased level of *Lactobacillus* may represent a microbial response for countering the mucosal/epithelial damage caused by *Cryptosporidium* infection. Similarly, the increased levels of *Lactobacillus* may represent a microbial response for countering the mucosal/epithelial damage caused by *Cryptosporidium* infection. Recent studies by Charania et al. [30] also showed that in the mice not treated with antibiotics, *Lactobacillus* populations increased in the mice infected by *Cryptosporidium*, but decreased in the mice pre-treated with antibiotics such as cloxacillin.

However, it should be noted that in the current study, the mice did not show clinical signs of cryptosporidiosis, making them possibly asymptomatic carriers of the parasite. However, in other studies involving goats, which showed mild to severe clinical symptoms of cryptosporidiosis (diarrhoea, hypothermia, growth retardation, and mortality), bacterial species associated with SCFA production have been shown to be depleted, thereby impacting overall SCFA biosynthesis pathways [31].

Reportedly, lactate metabolising bacteria are highly active in the production of D-amino acids [32] and provide an elevated microbial response to balance the mucosal/epithelial damage caused by *Cryptosporidium* infection [33]. D-amino acids are known to promote crosstalk between the microbiome and host via binding to epithelia and immune cells [34,35]. D-amino acid-producing species, such as *Lachnospiraceae*, *Lachnoclostridium*, *Lactobacillus*, and *Marvinbryantia* spp., have been reported in the mouse colon [34,35]. However, our observations indicate that during cryptosporidiosis, D-amino acids were generated at greater levels by the microbiome in the small intestine. The elevated levels of *Lactobacillus, Lachnoclostridium* and *Lachnospiraceae*, especially in the duodenum and jejunum of infected mice, indicate a possible role of these bacteria in D-amino acid production. Our observations were in line to the observations of Sasabe et al. [35].

We found that the citrate cycle was more active across the intestine during cryptosporidiosis. *C. parvum* reportedly lacks the machinery for the citrate cycle pathway and requires salvaging of these metabolites in the host gut [36]. The current study shows, for the first time, a greater role of yeasts in this salvaging process and in driving the proxy-citrate pathway for *Cryptosporidium* (Figure 7). Microbial carboxylase transporter proteins have been shown to induce pathogenicity and colonisation of bacteria such as *Haemophilus influenzae* and *Salmonella enterica*. This activity uses energy sources, such as glutamate, and causes increased levels of dicarboxylic acids, for instance, acetate or hexanoate (Figure 4) [37]. *Cryptosporidium* excystation in the duodenum has been documented [38] and may be responsible for the observed upregulation of proteins associated with glycolysis, glutaminolysis, and the citrate cycle in the small intestine.

Compared to the small intestine, glutamine/glutamate metabolism was upregulated in the infected caecum. Glucose depletion in the caecum and the colon is known to trigger glutamate utilisation as the primary carbon source, by both *Cryptosporidium* and host defence cells [39,40]. In the context of cryptosporidiosis, we observed glutamate utilisation that is typical of parasitic activity for generating α-ketoglutarate, catalysed by glutamine synthetase, glutamate kinase, and glutamate-5-semialdehyde dehydrogenase, as previously documented [39].

We detected upregulated host and yeast transketolases, followed by yeast polyubiquitin proteins, indicative of these proteins/enzymes catalysing ubiquinone biosynthesis in the jejunum–ileum tract. The preliminary step of the ubiquinone biosynthesis pathway begins with E4P metabolism and is catalysed by glucose-6-phosphate dehydrogenase (G6PDH) in trypanosomatid [41], *Plasmodium* [42], and *C. parvum* infections [43]. Enzyme activities of the host, yeasts, and the parasite suggest a host–parasite–microbiome association in the small intestine. This association may have compensated the deficient *Cryptosporidium* metabolic machinery for synthesising ubiquinone (coenzyme Q), which is a critical element of the electron transport chain. Such associations and their benefits to *Cryptosporidium* multiplication have been reported in aquatic systems [19] and neonatal mice gut dysbiosis [8]. High yeast ubiquitin-related activity, metabolised by the ubiquitin transfer or conjugating enzymes, was observed across the small intestine, especially in the ileum. These proteins are required for the synthesis, transfer, and metabolism of ubiquinone [39,42]. While it has been predicted that *Cryptosporidium* salvages the host ubiquinone system [39], our study suggests significant salvaging of this system from the yeast population, which drives its upregulation during infection. More research is required to confirm this observation.

Host actin has been shown to be essential for the invasion and replication of apicomplexan parasites such as *Cryptosporidium* [44]. Previous cell culture and microscopy studies have showed that *Cryptosporidium* forces the host actins to assemble and polymerise into plaque structures in order to complete the invasion process by sporozoites [45,46]. However, upregulation of actin as a result of parasite infection has not been previously reported. In the context of cryptosporidiosis, upregulated actin expression in the mouse gut is a novel aspect which requires further elaboration through targeted proteomic studies.

### 3.2. Extra-Intestinal Effects of Cryptosporidiosis

In non-gut organs such as the liver, oxalic acid upregulation is indicative of likely hyperoxaluria or a hyperoxaluria-like condition. In this condition, glyoxylate metabolism is negatively affected due to the deficiency of hepatic alanine glyoxylate aminotransferase (AGT) and cytosolic glyoxylate reductase (GR) [47]. However, the relationship to hyperoxaluria as an indirect effect of *Cryptosporidium* infection in the gut remains to be determined.

Mitochondrial pyruvate carboxylase was possibly one of the most interesting of the expressed proteins in the liver. This zinc-containing protein, in the presence of allosteric activators such as acetyl-CoA, catalyses the ‘pyruvate → oxaloacetate’ reaction towards both Krebs cycle replenishment and gluconeogenesis [48]. However, excessive accumulation of oxalate (caused by oxaloacetate accumulation) in the liver of *Cryptosporidium*-infected mice may be attributed to the greater expression of L-lactate dehydrogenase (LDH). The role of hepatic LDH in converting glyoxylate to oxalate has recently been reported for primary hyperoxaluria mouse models [49] and blood-based protozoal infections such as that with *Plasmodium* [50]. However, its indirect hepatic activity, especially as a follow-up pyruvate carboxylase activity, due to gut infection has not been reported and its dynamics require further study.

Interestingly, an increased expression of β-haemoglobin was seen in hepatic and serum matrices (Table S12). Buffalo [51] and clinical models [52] have shown elevated blood haemoglobin during cryptosporidiosis, similar to that observed in the current study. The correlation between iron uptake and gut microbiota during gut disorders has been highlighted [53], but more studies are required to better understand this interaction.

In summary, the murine study herein helped us to understand the biochemical phenomenon of *Cryptosporidium* infection across different parts of the gut and in non-gut organs. However, it should be noted, due to the differences between human and mouse gut systems, further studies must be performed to extrapolate these biochemical changes to cryptosporidiosis in clinical models. Furthermore, the immunopathology experiments combined with multi-omics platforms would also be able to better clarify the similarities and differences between human and germ-free mouse models.

## 4. Materials and Methods

### 4.1. Animal Ethics and Husbandry

All experiments were approved by the Monash University Animal Ethics Committee (Monash University AEC no. MARP/2018/055) following the guidelines of the Victoria State Government and the National Health and Medical Research Council, Australian Government. Mice were housed in Optimice cages containing sterile sawdust at 18–24 °C, 40–70% humidity, and 12:12 h light/dark cycle. Mice were provided with sterile water and feed (Ridely AgriProducts Pty. Ltd., Melbourne, VIC, Australia) ad libitum.

### 4.2. Mouse Infection Model

**Figure 8:**
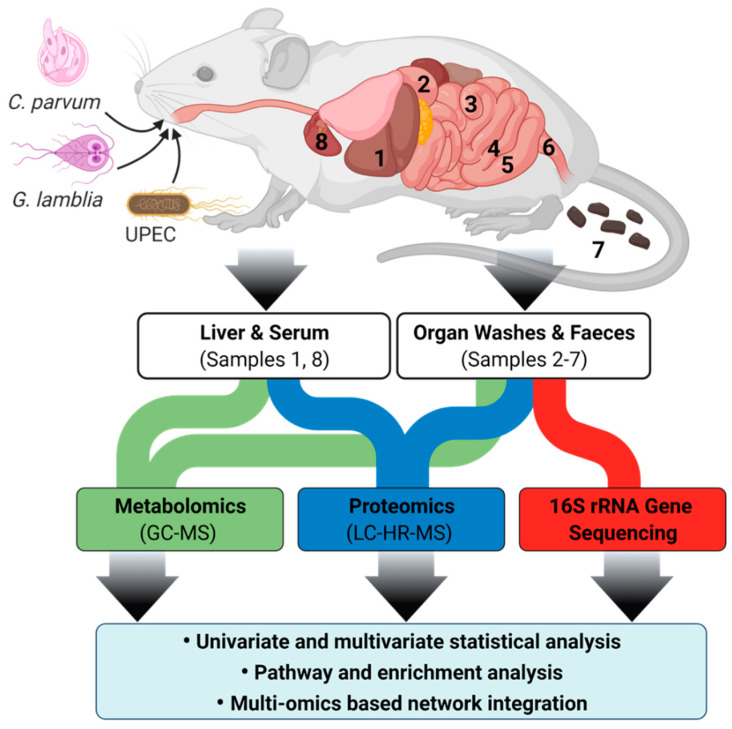
Overview of mouse cryptosporidiosis interaction study design showing various mouse samples that were collected. Samples were subjected to GC-MS and LC-HR-MS and resulting data were analysed by multivariate statistics. Samples are annotated as (1) liver tissue, washes of (2) duodenum, (3) jejunum, (4) ileum, (5) caecum, (6) colon, (7) faeces, and (8) serum.

The C57BL/6J strain was selected for this study, based on the results of a 14-day pilot study where *C. parvum* infection was compared in Balb/C, C57BL/6J and Swiss mice. For the main study, groups of five, 3-week-old C57BL/6J female mice were acclimatised for one week before infection with either 1 × 10^5^ *C. parvum* or *G. lamblia* oocysts (*C. parvum*, Cat. Number: C10E7; *G. lamblia*, cat. Number: G10E6; BTF Pty Ltd., North Ryde, NSW, Australia) or 1 × 10^8^ CFU of UPEC (ST131 lineage strain EC958) via oral gavage. An additional group of uninfected mice (*n* = 5) was also included for comparison against the infected groups. The minimum number of mice per group was based on the criteria set by the Metabolomics Standards Initiative [54,55]. *Cryptosporidium* and *Giardia* infection was monitored for 10 days via daily faecal collection and detection of oocysts by fluorescent microscopy using the EasyStain kit^TM^ (Biopoint Pty Ltd., Sydney, Australia) following the ISO 15553:2006 protocol [56] (Supplementary Materials Section 1.1). UPEC infection was quantified using a previously described method [21]. Mice were euthanised at 10 dpi by CO_2_ exposure and liver tissue, serum, and faeces collected. The luminal contents of the duodenum, jejunum, ileum, caecum, and colon were sampled by flushing 1.0 mL of sterile phosphate buffer saline through each section of the gut and collecting the contents (Figure 8) (Note: individual sectional length determined by the previous studies [57,58]). Serum (collected by cardiac bleed) and liver tissue samples were used as representatives for indirect and cross-organ effects of *C. parvum* infection. The luminal contents represented the direct effects of infection. All samples were immediately stored on dry ice and then at −80 °C until further analysis.

### 4.3. Untargeted Metabolomics by Gas Chromatography-Mass Spectrometry (GC-MS)

For untargeted metabolomics analysis, samples were prepared as previously described [59], with minor modifications. Briefly, frozen samples of faecal pellets (10–25 mg, wet weight), serum (10–15 mg, wet weight), liver (40–50 mg, wet weight), and duodenum, jejunum, ileum, caecum, and colon washes (200–250 mg, wet weight) were transferred to 1.5 mL homogenisation tubes (Navy RINO lysis kit, BioTools Pty. Ltd., Keperra, QLD, Australia). Frozen feacal samples were used following previous protocols [60,61]. These were chosen over freeze drying as the water content variance in the mice fecal samples was negligible across the sampled mice, and to minimize analyte losses that have been reported in other studies post freeze drying [62]. A 1 mL aliquot of chilled extraction solution (−20 °C) comprising acetonitrile, isopropanol, and water (3:3:2, *v/v/v*) spiked with “Internal Standard 1” (Valine^13^C_2_ and Stearic acid^13^C, both 10 µg; Novachem Pty. Ltd., Heidelberg West, VIC, Australia) was then added to each of the sample tubes. The samples were then homogenised at 6800 rpm for 2 × 20 sec cycles, with 10 sec rest, at room temperature (Percelleys Evolution, Bertin Instruments, Montigny-le-Bretonneux, France). The homogenised samples were centrifuged at 14,000× *g* for 2 min at 4 °C. A 100 µL aliquot of the supernatant was transferred to a glass vial with fused inserts. The samples were dried in a vacuum centrifuge at 37 °C. Upon drying, 50 µL Myristic acid-d27 (Sigma Aldrich, St. Louis, MO, USA; 0.2 mg/mL in methanol) was added as “Internal Standard 2”. The samples were re-dried in a vacuum centrifuge. The samples were derivatised “in-time”, followed by a 1-h holding time, before injection into a GC-MS as previously reported [63,64].

A quality control (QC) mix containing 19 different polar and semi-polar metabolites was prepared as per Fiehn, O. [59] (Supplementary Materials). The QC mix samples were subjected to derivatisation and injection, as indicated above, at a rate of 1 QC sample per 15 samples. Raw data, obtained from 7 batches processed on the MassHunter workstation, were subjected to the batch effect adjustment tool of MetaboAnalyst 4.0 [65]. The batch effect adjusted data were further normalised to the IS2 (Myristic acid-d27, 10 µg per sample, relative standard deviation (RSD) = 9.21%). Similarly, variability between the samples was indicated by the RSD of IS1 (Valine-^13^C_2_ = 7.86% and Stearic acid-^13^C = 1.87%). Additionally, the metabolic output was further normalised according to the sample weights and was expressed as metabolite concentration (µg/g wet sample weight).

### 4.4. Metaproteome Extraction and Proteome Analysis

Proteomics samples comprised serum and liver tissues (wet weight = 50 ± 2.5 mg), 250 mg of luminal contents (duodenum, jejunum, ileum, caecum, and colon), and faeces (20 mg). Samples were weighed and transferred to 1.5 mL bead mill homogeniser tubes. Urea (8 M, in 20 mM Tris, pH 8, 50 µL) was added to all samples except for luminal content samples. Samples were homogenised at a frequency of 28 s^−1^ for 3 × 10 min in a Qiagen TissueLyzerII system (Qiagen Pty Ltd., Chadstone, VIC, Australia). Samples were centrifuged (16,000× *g*, 5 min, 4 °C), and the supernatant transferred to new tubes. Room temperature MilliQ water (50 µL) was added, followed by cold acetone (−20 °C, 400 µL). The samples were incubated at −20 °C for 1 h and centrifuged (16,000× *g*, 5 min, 21 °C). The supernatant was decanted, and the pellet was re-washed with 200 µL acetone, followed by re-centrifuging and air-drying. Samples were incubated in 8 M urea for 1 h, followed by water bath sonication (room temperature, 10 min) and centrifugation (16,000× *g*, 5 min, 21 °C). The supernatant was transferred to a fresh tube.

Tryptic peptides (100 ng) were desalted and concentrated with a trap column (PepMap100 C18 5 mm × 300 µm, 5 µm) and separated on a nano column (PepMap100 C18 150 mm × 75 µm, 2 µm) using an UltimateTM 3000 RSLC nano-LC system, with mobile phases (A: water + 0.1% (*v/v*) formic acid; B: acetonitrile (80% *v/v*) + 0.08% (*v/v*) formic acid). The peptides were eluted using Solvent B at gradients of 5–40% (0–60 min) and 40–99% (60–70 min). The eluted peptides were ionized with a Nanospray Flex Ion Source (Note: All instruments and parts of Liquid Chromatography-High resolution mass spectrometry (LC-HR-MS) were sourced from Thermo Scientific Australia Pty Ltd., Scoresby, VIC, Australia). The Protein Discoverer 2.2 (Thermo Scientific) and Sequest HT search engines were used to identify peptides/proteins and quantify the relative abundance of proteins (Further details are provided in the Supplementary section).

To analyse protein expression across the intestine, data from individual sections of the small intestine and large intestine were, respectively, combined in the Biomarker meta-analysis tool of Metaboanalyst 4.0. The output was obtained as combined Log_2_Fold change (cFC) with the minimum cut-off of cFC = 1 and *p*-value (FDR adjusted) ≤ 0.05.

### 4.5. Genomic Extraction, Analysis and Processing

Mouse faeces and luminal contents (*n* = 5 each) were homogenised and DNA was extracted using the manufacturer’s instructions (ZymoBiomics DNA miniprep kit, Zymo Research Corp., Irvin, CA, USA). Amplicons were generated from the V3 and V4 regions of 16S rRNA using gene-specific primers (in bold) 515f (5′-*TCGTCGGCAGCGTCAGATGTGTATAAGAGACAG***GTGCCAGCMGCCGCGGTAA**-3′) and 806rbc (5′-*GTCTCGTGGGCTCGGAGATGTGTATAAGAGACAG***GGACTACHVGGGTWTCTAAT**-3′) (Integrated DNA Technologies, Inc., Coralville, IA, USA) with the appropriate adapter sequence for Illumina sequencing (in italics).

Amplicon products were purified and quantified before being sequenced and demultiplexed on an Illumina MiSeq using a v3 300 bp PE sequencing kit following the manufacturer’s protocol (see Supplementary Materials for further details). Sequence analysis was performed using QIIME 2 (Release no. 2019.7) pipeline [66] against the Greengenes database, as previously described [17]. The sequencing efficiency was determined by comparing the percentage of different OTUs identified in the microbial community standard II sample (Log distribution) (ZymoBiomics D6310, Zymoresearch Corp., Irvine, CA, USA) with the manufacturer’s data. Multivariate statistics using METAGENassist analysis [67] were performed to investigate the metabolic nature of the microbial community detected in each sample group.

### 4.6. Multi-Omics Integration and Statistical Analysis

The metabolomics and proteomics data were adjusted for batch-effect, log transformed and multivariate data analysis conducted with the software SIMCA (version 16, Sartorius Stedim Biotech, Umeå, Sweden) and MetaboAnalyst 4.0 [65]. The cut-off level for significant metabolites was a signal-to-noise (S/N) ratio of 10, while for proteins, it was a relative abundance of 1 × 10^5^. For statistical analysis of both metabolome and proteome, a fold change of ≤0.5 (downregulation) or ≥2.0 (upregulation), and a Benjamini–Hochberg adjusted *p*-value of ≤0.05 were set as the minimum cut-off threshold levels. Metabolic and proteomic outputs were integrated using the “Joint-pathway analysis tool” of MetaboAnalyst 4.0 and Paintomics 3 [68]. The metabolic pathway networks obtained after statistical analyses were manually curated in Omix visualization software (Version 1.9.34; Omix Visualisation GmbH and Co. KG, Lennestadt, Germany).

## 5. Conclusions

We utilised a mouse model to study the direct (gut) and indirect (serum and liver) effects of cryptosporidiosis using a multi-omics approach. Energy pathways such as glycolysis and glutaminolysis were significantly impacted in the jejunum and ileum during cryptosporidiosis. The proteomic and metabolic outputs indicated an underdeveloped proxy-citrate cycle in *Cryptosporidium*, partially salvaged from the host gut, with additional input of yeast enzymes. Instead of the commonly reported G6PDH-catalysed route, the ubiquinone (CoQ) biosynthesis system in the ileum appeared to begin with host transketolase activity, followed by the salvaging of the yeast ubiquinone biosynthesis system by the parasite. The gut microbiome response to cryptosporidiosis was detected via increased levels of D-amino acids and SCFAs. Similarly, high oxalate accumulation in the liver indicated enteric hyperoxaluria as a likely indirect effect of cryptosporidiosis. Our study shows the ability of multi-omics to contribute a robust understanding of gut infections and demonstrates the previously unreported infection interactomics as the parasite passes through the gut, as well as how these interactomics have effects beyond the gut. These results provide a platform from which new avenues of precision medicine and improved treatment methods for cryptosporidiosis may be devised.

## Figures and Tables

**Table 1:** Most significant metabolic pathways in the gut modulated during cryptosporidiosis with respect to the uninfected mice, based on integration of the metabolomics and proteomics data using a joint pathway analysis tool.

Metabolic Pathway	Match Status	Impact	FDR
Arginine biosynthesis	13/27	1.12	<0.0001
Citrate cycle (TCA cycle)	15/42	1.95	<0.0001
Glycolysis or Gluconeogenesis	16/61	1.28	<0.0001
Pyruvate metabolism	13/45	0.93	<0.0001
Nitrogen metabolism	6/10	1.00	0.0002
Glutathione metabolism	13/56	0.69	0.0006
Alanine, aspartate, and glutamate metabolism	13/61	0.83	0.0015
Glyoxylate and dicarboxylate metabolism	12/56	0.53	0.0023
Galactose metabolism	11/51	0.66	0.0035
Arginine and proline metabolism	14/78	0.52	0.0039

Note: Match status = number of (significant metabolites and proteins/total metabolites and proteins) in a pathway; FDR = false discovery rate.

## Data Availability

The data presented in this study are available on request from the corresponding author. The data are not publicly available due to intellectual property restrictions.

## Supplementary Materials

The following are available online at https://www.mdpi.com/article/10.3390/metabo11060380/s1. Supplementary Materials: The metadata and other data from 16S rRNA sequencing, proteomics and metabolomics outputs including pathways and pathway impact are presented in this document. Supporting Dataset 1: This dataset represents the microbial protein expression across different sections of mouse intestine during cryptosporidiosis. The expression is shown by combined Log_2_(Fold change) in an infected mouse with respect to its uninfected counterpart. Supporting Dataset 2: This dataset represents the host protein expression across different sections of mouse intestine during cryptosporidiosis. The expression is shown by combined Log_2_(Fold change) in an infected mouse with respect to its uninfected counterpart. Supporting Dataset 3: This dataset represents the host protein expression across different extra-intestinal sections of mouse (serum and liver) during cryptosporidiosis. The expression is shown by combined Log_2_(Fold change) in an infected mouse with respect to its uninfected counterpart.
