# Supplementary material for: Cryptosporidiosis Modulates the Gut Microbiome and Metabolism in a Murine Infection Model
Source: Metabolites. 2021 Jun 11;11(6):380. doi: 10.3390/metabo11060380 (PMC8230837; doi:10.3390/metabo11060380)
Supplement: Supplementary file 1 [file metabolites-11-00380-s001.zip › Supplementary materials_Cryptosporidium Interactomics_Metabolites_Final copy.pdf]

Supplementary Materials

Cryptosporidiosis modulates gut microbiome metabolism and the immune response in an infected host.

Avinash V Karpe[1], Melanie L Hutton[2], Steven J Mileto[2], Meagan L James[2], Chris Evans[2], Rohan M Shah[1,3], Amol B Ghodke[4], Katie E Hillyer[1], Suzanne S Metcalfe[1], Jian-Wei Liu[5], Tom Walsh[5], Dena Lyras[2*], Enzo A Palombo[3*], David J Beale[1*]

[1]Land and Water, Commonwealth Scientific and Industrial Research Organization, Ecosciences Precinct, Dutton Park, QLD 4102, Australia.

[2]Infection and Immunity Program, Monash Biomedicine Discovery Institute and Department of Microbiology, Monash University, Clayton, VIC 3168, Australia.

[3]Department of Chemistry and Biotechnology, Faculty of Science, Engineering and Technology, Swinburne University of Technology, Hawthorn, VIC 3122, Australia.

[4]Queensland Alliance for Agriculture and Food Innovation, Department of Horticulture, The University of Queensland, St Lucia, QLD 4072, Australia. & BIO21 Institute, School of Biosciences, The University of Melbourne, Parkville, VIC 3010, Australia

[5]Land and Water, Commonwealth Scientific and Industrial Research Organization, Agricultural and Environmental Sciences Precinct, Acton, Canberra, ACT 2601, Australia.

* These authors contributed equally and should be regarded as joint corresponding authors

1. Materials and methods

1.1. Metabolome extraction and analysis

The homogenised samples prepared for metabolomic analysis were subjected to in-time derivatisation on an Agilent 7890B gas chromatography system with 5977B mass spectrometry detector fitted with an MPS autosampler (Gerstel GmbH & Co. KG, Mülheim an der Ruhr, Germany). Methoxyamine HCl (MOX) (20 mg/mL in pyridine) and N,O-Bis(trimethylsilyl)trifluoroacetamide (BSTFA) were added to the samples in 20 μL and 40 μL volumes, respectively, by the autosampler. The samples were incubated at 37°C for 60 min and 30 min after the addition of MOX and BSTFA, respectively. After a 60-minute wait-time, samples were injected to the GC-MS system with the previously reported configurations and settings 18.

1.2. Quality control mix

A quality control (QC) mix containing 19 different polar and semi-polar metabolites was prepared as per Fiehn (2016). The QC mix consisted of 500 ng dried metabolites of valine, succinic acid, methionine, 4-hydroxyproline, salicylic acid, α-ketoglutaric acid, shikimic acid, citric acid, lysine, glucose, sucrose, chlorogenic acid, myristic acid, myristic acid-d27, stigmasterol, glucose ($U^{13}C_6$), L-glutamine (Amide-^{15}N), palmitic acid (1-^{13}C) and glycine (1-^{13}C).

The metabolomics data, after Log_{10} normalisation, were then analysed using multivariate data analysis software SIMCA (version 16, Sartorius Stedim Biotech, Umeå, Sweden). Principal component analysis (PCA), the unsupervised model, was initially undertaken to find statistically significant differences between sample groups. This was followed by partial least square discriminate analysis (PLS-DA).

The PCA and PLS-DA model validity was determined by R^2X, R^2Y and Q^2 values. The R^2X and R^2Y values define variation between X and Y variables of various components in the sample set and, Q^2 gives predictability of the model [22]. Furthermore univariate statistical tools such as volcano plots, enrichment analysis and pathway impacts were produced by MetaboAnalyst 4.0 [19]. The cut-off level for significant metabolites was a signal-to-noise (*S*/*N*) ratio of 50, a fold change of ≤ 0.5 (downregulation) or ≥ 2.0 (upregulation) and a Benjamini–Hochberg adjusted p-value of ≤ 0.05.

1.3. Metaproteome extraction, LC-MS/MS analysis and data processing

After the homogenisation and extraction, the tryptic peptides (100 ng) were desalted and concentrated with a trap column (PepMap100 C18 5 mm × 300 μm, 5 μm) and separated on a nano column (PepMap100 C18 150 mm × 75 μm, 2 μm) using an Ultimate™ 3000 RSLC nano LC system, with mobile phases (A: water + 0.1% (*v*/*v*) formic acid; B: acetonitrile (80% *v*/*v*) + 0.08% (*v*/*v*) formic acid). The

peptides were eluted using Solvent B at gradient s of 5 - 40% (0 - 60 min) and 40 - 99% (60 - 70 min). The eluted peptides were ionized with a Nanospray Flex Ion Source. The spray voltage was set to 2.3 kV and temperature of the heated capillary was set at 300°C. After ionization, mass spectra (MS1) and tandem mass spectra (MS/MS) analysis was performed using an Orbitrap Fusion MS. The MS survey scans of peptide precursors were performed in the Orbitrap detector and the scan range was 400 to 1500 *m/z* at resolution of 120 K (at 200 *m/z*). The target value of automatic gain control (AGC) was set as 4×10^5. The maximum injection time for the MS was 50 ms. MS/MS was performed on the most abundant precursors of charge states 2+ to 7+ with intensity greater than 1×10^5. They were isolated by the quadrupole with a window of 1.6 *m/z*. Fragmentation was achieved by high-energy collisional dissociation (HCD) with collision energy of 28%. Fragments were detected in the ion trap detector in rapid scan rate mode. The AGC target was 4×10^3, maximum injection time was 300 ms and the dynamic exclusion was 15 seconds. The instrument was set to run in top speed mode with a 3 second cycle for both the MS and MS/MS scans (***Note:*** *All instruments and parts of Liquid chromatography-High resolution mass spectrometry (LC-HR-MS) were sourced from Thermo Scientific Australia Pty Ltd, Scoresby, VIC, Australia*).

Protein Discoverer 2.2 (Thermo Scientific) and Sequest HT search engine were used to identify peptides/proteins and quantify relative abundance of proteins. The spectrum data was searched against the UniProt databases indicated below. Precursor mass tolerance was set to 10 ppm and product ions were searched at 0.6 Da. Three missed tryptic cleavages were allowed. Modification included Oxidation (+ 15.995 Da), Deamidation (+ 0.984 Da), Amidation (- 0.984Da), and Propionamidation (+ 71.037 Da). Peptide spectral match was validated using the Percolar algorithm, based on q-values and 1% False Discovery Rate (FDR). Relative abundance was calculated from precursor abundance intensity, normalized to total peptide amount (Ratio calculation based on summed abundance; ANOVA test based on individual proteins).

The proteomics data, after Log_{10} normalisation, were then analysed using multivariate data analysis software SIMCA (version 16, Sartorius Stedim Biotech, Umeå, Sweden). Principal component analysis (PCA), the unsupervised model, was initially undertaken to find statistically significant differences between sample groups. This was followed by partial least square discriminate analysis (PLS-DA). Uniprot databases used were *Mus musculus* (UP000000589) and various microbial databases (UP000242190, P000001218, UP000280657, UP000005219, UP000003081, UP000004459, UP000004596, UP000018901, UP000263263, UP000029920, UP000315402, UP000000927, UP000006599, UP000027129, UP000064844, UP000008178, UP000000625, UP000255233, UP000309428, UP000012589, UP000001377, UP000001415, UP000006000, UP000285063, UP000000439, UP000005561, UP000017429, UP000005384, UP000092574, UP000195817, UP000001031, UP000006657, UP000000333, UP000002938, UP000000559,

UP000270830, UP000236311, UP000002037, UP000002311, and UP000006726) based on the genomic output.

The PCA and PLS-DA model validity was determined by R^2X, R^2Y and Q^2 values. The R^2X and R^2Y values define variation between X and Y variables of various components in the sample set and, Q^2 gives predictability of the model [22]. Furthermore univariate statistical tools such as volcano plots, enrichment analysis and pathway impacts were produced by MetaboAnalyst 4.0 [19]. The cut-off level for significant proteins was a fold change of ≤ 0.5 (downregulation) or ≥ 2.0 (upregulation) and a Benjamini–Hochberg adjusted p-value of ≤ 0.05.

1.4. Genomic extraction, analysis and processing

Mouse faeces (13-32 mg) and intestinal washes (50 µl) of duodenum, jejunum, ileum, caecum and colon samples (all, n = 5) were homogenised for 4 cycles of 6,800 rpm × 20 s (Precellys® Evolution, Bertin Technologies, France) and extracted using the ZymoBiomics DNA miniprep kit (Zymo Research Corp., Irvin, CA, USA) following the manufacturer's instructions.

Amplicons were generated from the V3 and V4 regions of 16S rRNA using gene-specific primers (in bold) with the appropriate adapter sequence for Illumina sequencing (in italics)
515f (5'- *TCGTCGGCAGCGTCAGATGTGTATAAGAGACAG***GTGCCAGCMGCCGCGGTAA** -3') and
806rbc (5'-*GTCTCGTGGGCTCGGAGATGTGTATAAGAGACAG***GGACTACHVGGGTWTCTAAT** -3') (Integrated DNA Technologies, Inc., Coralville, IA, USA). Pooled DNA extracts were quantified (Qubit 3.0, ThermoFisher Scientific), and triplicate samples amplified using the following reaction mix: 10 ng DNA, 10 µl Platinum™ Hot Start PCR 2x Mastermix (Invitrogen™, Carlsbad, CA, USA), 0.5 µl of each 10 µM primer and water added to make the volume to 25 µl. The PCR conditions were 94°C/2 min, followed by 35 cycles of 94°C/30 s, 50°C/30 s and 72°C/1 min, and a final elongation step at 72°C/5 min.

Amplicon products were purified with 1.8x volume of Agencourt® AMPure XP beads (Beckman Coulter™, Brea, CA, USA) and suspended in 50 µl elution buffer (Qiagen, Hilden, Germany). Illumina index PCR was conducted following the Illumina amplicon sequencing protocol (94°C/2 min, followed by 8 cycles of 94°C/30 s, 55°C/30 s and 72°C/1 min, and a final elongation step at 72°C/5 min). Amplified products were re-purified with 1.12*x* volume of AMPure XP beads, size assessed as 456 bp on a Tape Station (Life Technologies, Carlsbad, CA, USA) using the High Sensitivity DNA screen tapes, quantified (Qubit 3.0) and pooled in equimolar concentrations (4nM). The purified library was sequenced and demultiplexed on an Illumina MiSeq using a v3 300 bp PE sequencing kit following the manufacturer's protocol.

The 16s metagenomic analysis was of a pooled control and treatment sample to represent the maximum diversity present. Sequence processing was performed using QIIME 2 (Release no. 2019.7)

pipeline [20] similar to previously reported methodology [15]. Briefly, after checking the sequence quality 3 nucleotides were removed from left and right end of each sequence. The sequence length retained ranged from 284 – 319 bp with average 286 bp length. A total of 847 features were detected in all samples, represented by 65.39% retained sequences. Average feature count of the control sample group was 24188 and 24290 for sample treated with *Cryptosporidium* spiked diet. METAGENassist analysis [21] was performed to investigate the metabolic nature of the microbial community detected in each treatment group.

2. Results

During cryptosporidiosis, most metabolites were depleted (FC < 0.5) in the small intestine. Primary depleted metabolites in the duodenum (Figure 3, Tables S3 – S5) included succinate, 6-hydroxy caproate, adenosine and inosine monophosphates, and glucose. In the jejunum, maleate, non-hexose sugars, and sugar acids (glucoheptonate and sedoheptulose) were depleted (Figure 2, Table S4). In the ileum, depleted metabolites included sorbose, erythrose phosphate, ribitol, glycerol, and gluconate, while shikimate, phenaceturate, and urea were elevated (Figure 2, Table S5). Overall, during cryptosporidiosis, more metabolites, especially sugars, sugar acids, and sugar alcohols, were depleted in the small intestine than elevated. Changes in fatty acid metabolism, especially of medium-chain and long-chain fatty acids (MCFAs and LCFAs), were observed in the intestine during infection. Although fatty acid oxidation and glycerolipid metabolism was observed in the duodenum, the latter was more prominent throughout the small intestine, indicated by a significant decrease of glycerol in the jejunum (FC = 0.07) and the ileum (FC = 0.04), and palmitate (FC = 0.26) and, palmitoleate (FC = 0.22) in the ileum.

On the contrary, the number of elevated metabolites increased in the caecum and colon during infection. In the caecum, amino acids such as glycine, methionine, creatinine, tyrosine, alanine, lysine, and cysteine were elevated (Figure 2, Table S6). Other elevated metabolites in the colon included fatty and organic acids, such as malate, 3-aminoisobutyrate, fumarate, 3,4-dihydroxymandelate, and citrate (Figure 2, Table S7). Metabolic composition of the faeces was similar to that of the colon, with the addition of increased abundances of organic acids (Figure 2, Table S8).

Table S1. Statistical representation of data quality of 16S rRNA gene sequencing via the estimators of sequence diversity and richness across uninfected and *C. parvum* infected organ wash samples.

Experimental set	Sample ID	Average OTU count	Chao1	Ace	Shannon	Good's Coverage	Simpson
Uninfected	Faeces 0 dpi	88,250	185.00	185.17	7.12	1.000	0.99
	Faeces 10 dpi	155,743	156.33	156.33	6.38	1.000	0.98
	Duodenum	148,152	232.20	232.32	7.24	1.000	0.99
	Jejunum	162,266	151.00	151.00	6.54	1.000	0.98
	Ileum	163,879	159.00	159.17	6.52	1.000	0.98
	Caecum	100,420	187.25	187.33	6.65	1.000	0.98
	Colon	153,308	130.00	130.22	6.04	1.000	0.97
Cryptosporidiosis	Faeces 0 dpi	127,391	153.00	153.00	6.48	1.000	0.98
	Faeces – 10 dpi	112,841	132.50	131.68	6.04	0.999	0.98
	Duodenum	158,630	123.00	122.76	6.10	0.999	0.98
	Jejunum	154,824	188.14	188.51	6.99	1.000	0.99
	Ileum	134,650	191.00	190.63	7.19	0.999	0.99
	Caecum	106,048	322.33	322.30	7.95	1.000	0.99
	Colon	155,536	277.00	275.82	7.50	0.999	0.99

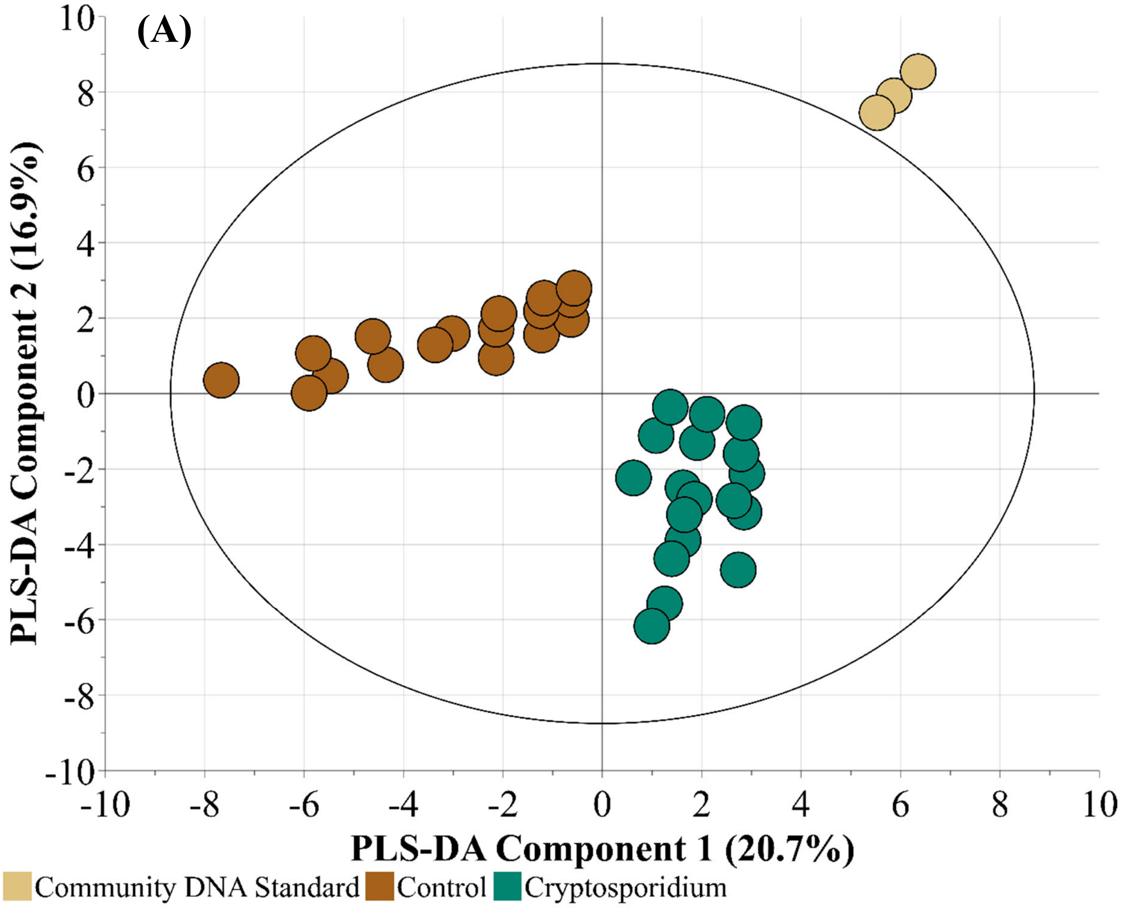

(A)
PLS-DA Component 2 (16.9%)
PLS-DA Component 1 (20.7%)
10
8
6
4
2
0
-2
-4
-6
-8
-10
-10
-8
-6
-4
-2
0
2
4
6
8
10
Community DNA Standard
Control
Cryptosporidium

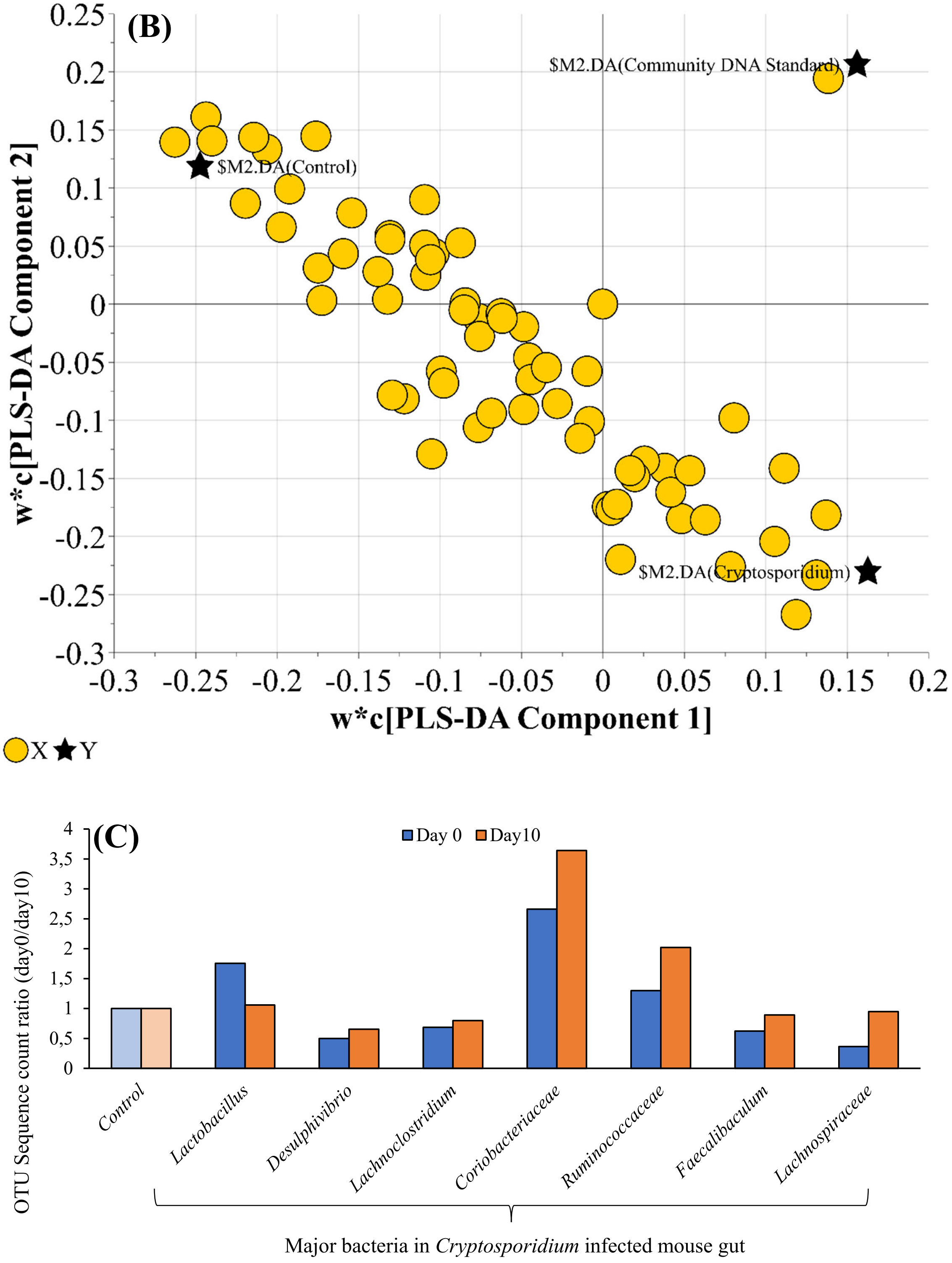

(B)
$M2.DA(Community DNA Standard)
$M2.DA(Control)
$M2.DA(Cryptosporidium)
w*c[PLS-DA Component 2]
w*c[PLS-DA Component 1]
0.25
0.2
0.15
0.1
0.05
0
-0.05
-0.1
-0.15
-0.2
-0.25
-0.3
-0.25
-0.2
-0.15
-0.1
-0.05
0
0.05
0.1
0.15
0.2
X Y
(C)
Day 0
Day10
OTU Sequence count ratio (day0/day10)
4
3,5
3
2,5
2
1,5
1
0,5
0
Control
Lactobacillus
Desulphivibrio
Lachnoclostridium
Coriobacteriaceae
Ruminococcaceae
Faecalibaculum
Lachnospiraceae
Major bacteria in Cryptosporidium infected mouse gut

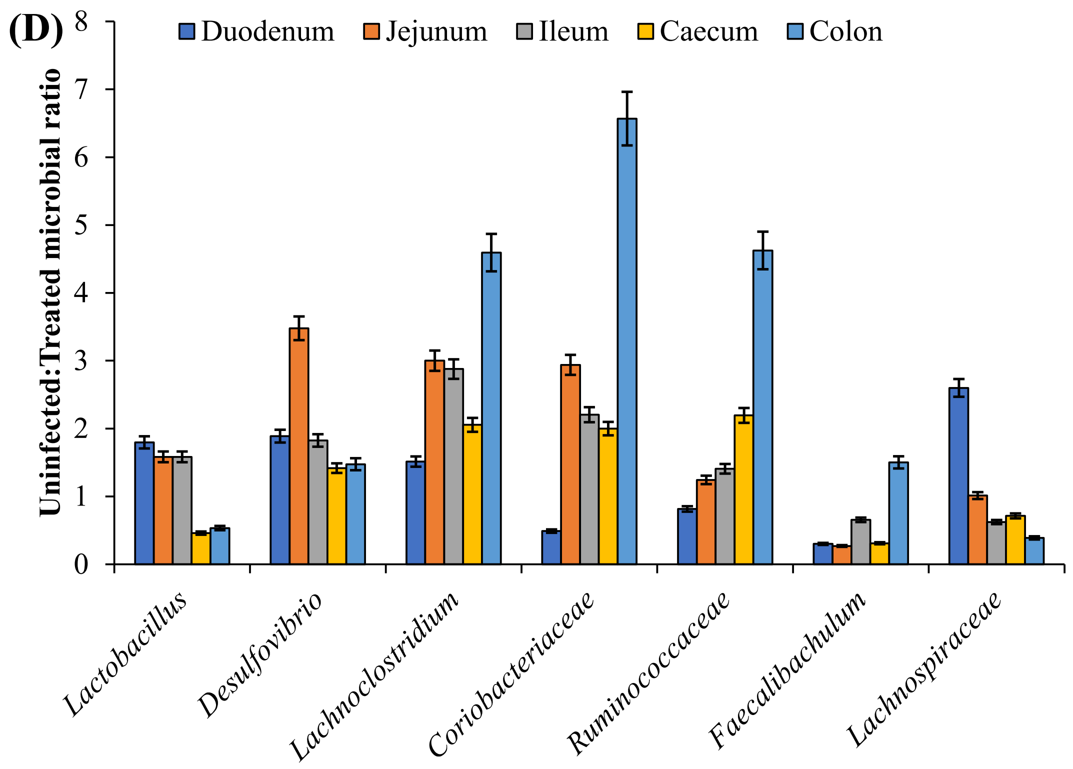
(D)
Duodenum
Jejunum
Ileum
Caecum
Colon
Uninfected:Treated microbial ratio
0
1
2
3
4
5
6
7
8
Lactobacillus
Desulfovibrio
Lachnoclostridium
Coriobacteriaceae
Ruminococcaceae
Faecalibachulum
Lachnospiraceae

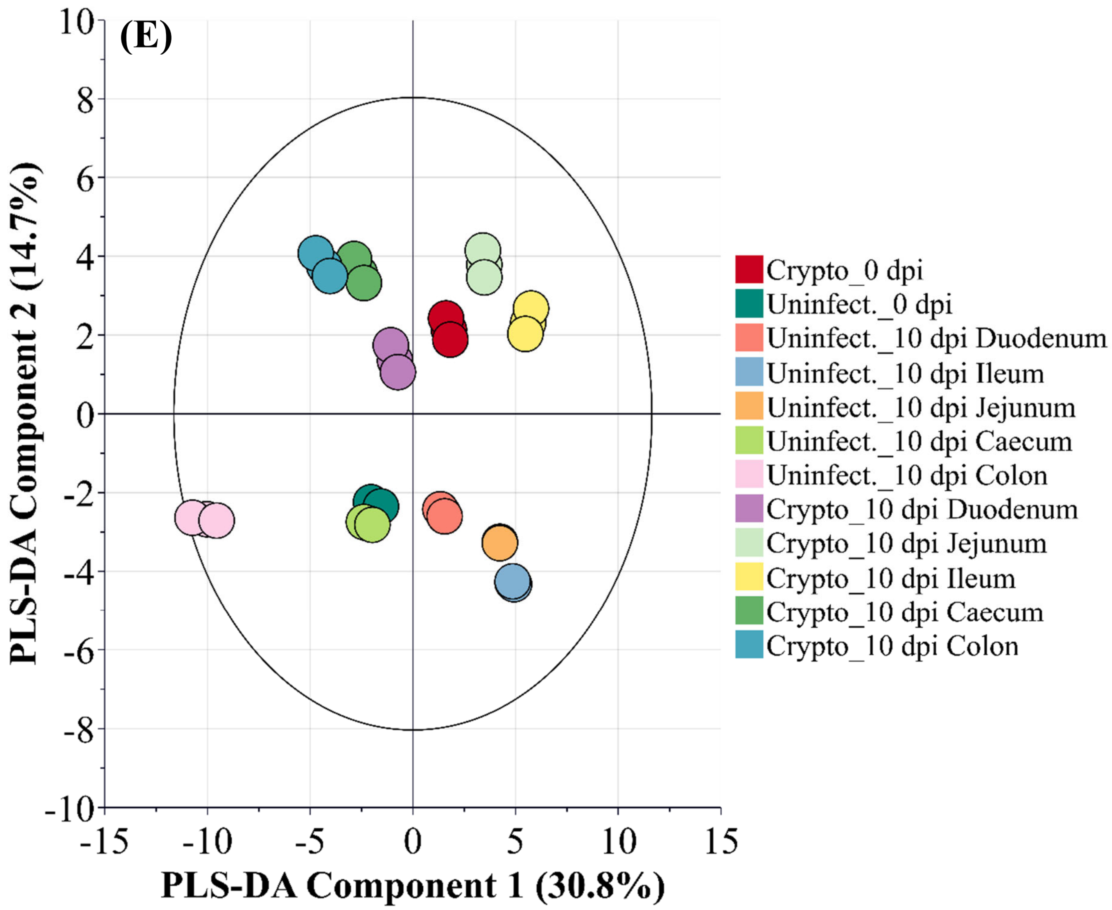
(E)
PLS-DA Component 2 (14.7%)
PLS-DA Component 1 (30.8%)
10
8
6
4
2
0
-2
-4
-6
-8
-10
-15
-10
-5
0
5
10
15
Crypto_0 dpi
Uninfect._0 dpi
Uninfect._10 dpi Duodenum
Uninfect._10 dpi Ileum
Uninfect._10 dpi Jejunum
Uninfect._10 dpi Caecum
Uninfect._10 dpi Colon
Crypto_10 dpi Duodenum
Crypto_10 dpi Jejunum
Crypto_10 dpi Ileum
Crypto_10 dpi Caecum
Crypto_10 dpi Colon

Figure S1. (A and B) PLS-DA score and loading scatter plots showing general discrimination between microbial spread across various sections of intestinal washes (R2X (cum) = 0.748, R2Y = 0.961, Q2 (cum) = 0.908) (Uninfect. = Uninfected samples, Crypto = *Cryptosporidium* infected' samples); (C) Comparison of microbial distribution between 0 dpi and 10 dpi, (D) Ratio of major genera showing prominent changes during cryptosporidiosis infection compared to the uninfected mice, in mouse intestine regions. (E) PLS-DA score scatter plot of intestinal washes ((R2X (cum) = 0.961, R2Y = 0.884, Q2 (cum) = 0.618))

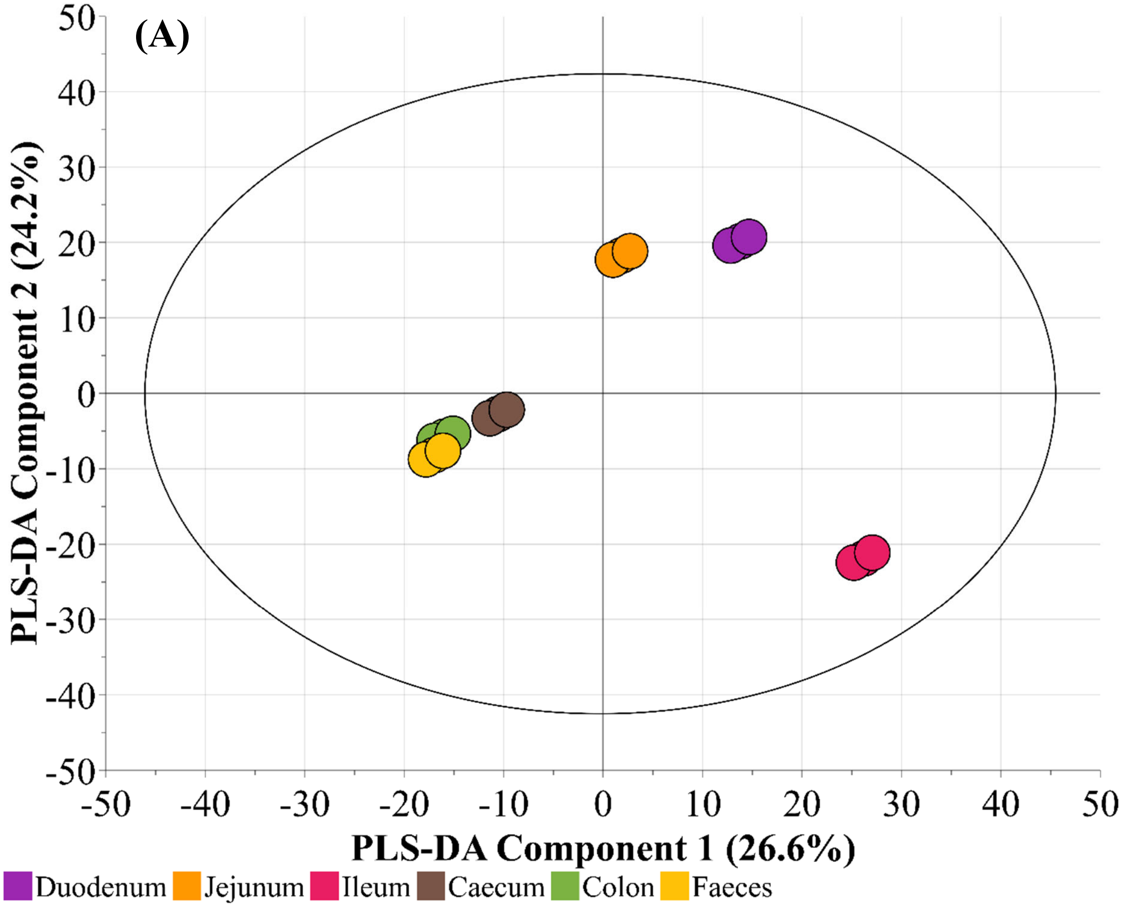
(A)
50
40
30
20
10
0
-10
-20
-30
-40
-50
PLS-DA Component 2 (24.2%)
-50
-40
-30
-20
-10
0
10
20
30
40
50
PLS-DA Component 1 (26.6%)
Duodenum
Jejunum
Ileum
Caecum
Colon
Faeces

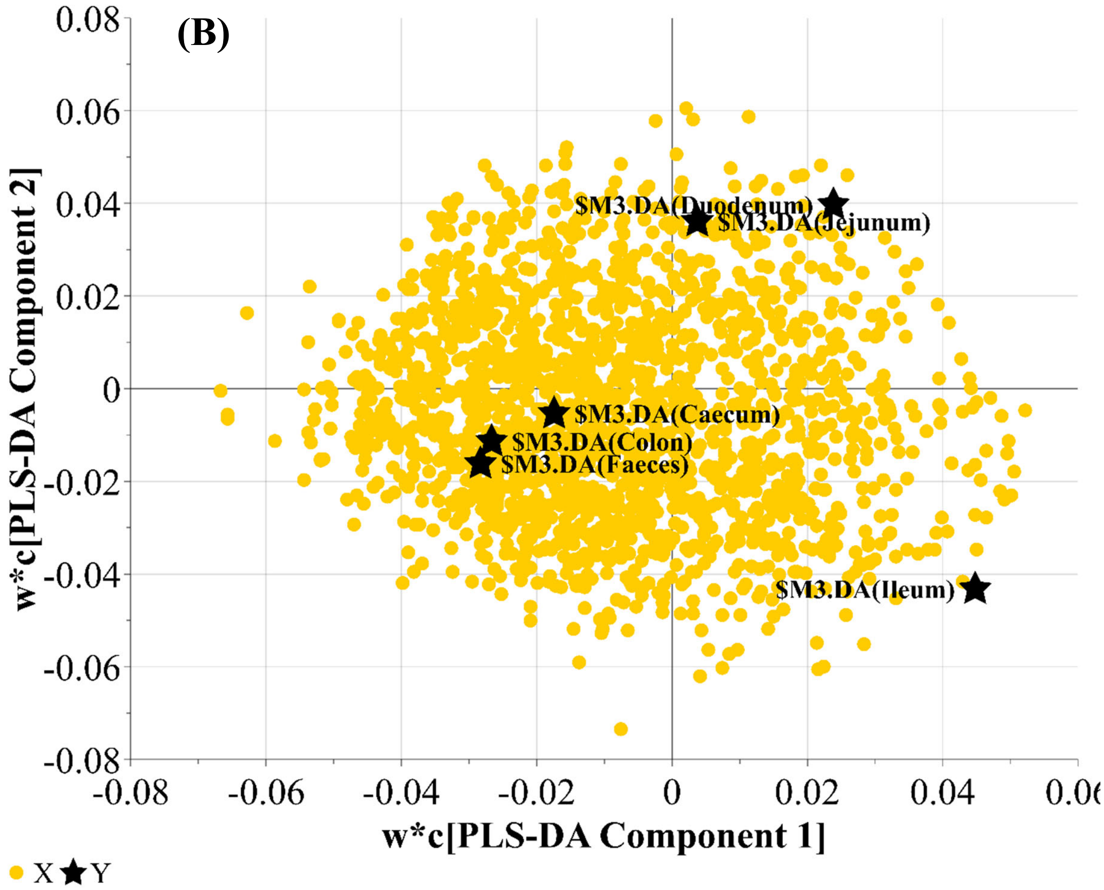

Figure S2. (A) PLS-DA score scatter plot and (B) PLS-DA loading scatter plots, for host protein expression in mouse gut during *Cryptosporidium* infection. R^2X (cum) = 0.88, R^2Y (cum) = 0.799, Q^2 (cum) = 0.408. Note: The ellipse (Figure S2A) indicated the area of 95% confidence interval as determined by Hotelling T^2 analysis.

Table S2. Model fit (R^2X and R^2Y) and predictability (Q^2) of metabolomic profile of all samples of *C. parvum* infected mice with respect to the uninfected mice, as analysed by the multivariate SIMCA analysis.

Sample	R^2X	R^2Y	Q^2
Faeces	0.877	0.998	0.93
Duodenum wash	0.664	0.999	0.834
Jejunum wash	0.822	0.999	0.909
Ileum wash	0.719	0.997	0.957
Caecum wash	0.528	0.998	0.890
Colon wash	0.703	0.991	0.917
Serum	0.755	0.99	0.928
Liver	0.881	0.99	0.794

Note: Further related information can be found in Figures S1 – S7.

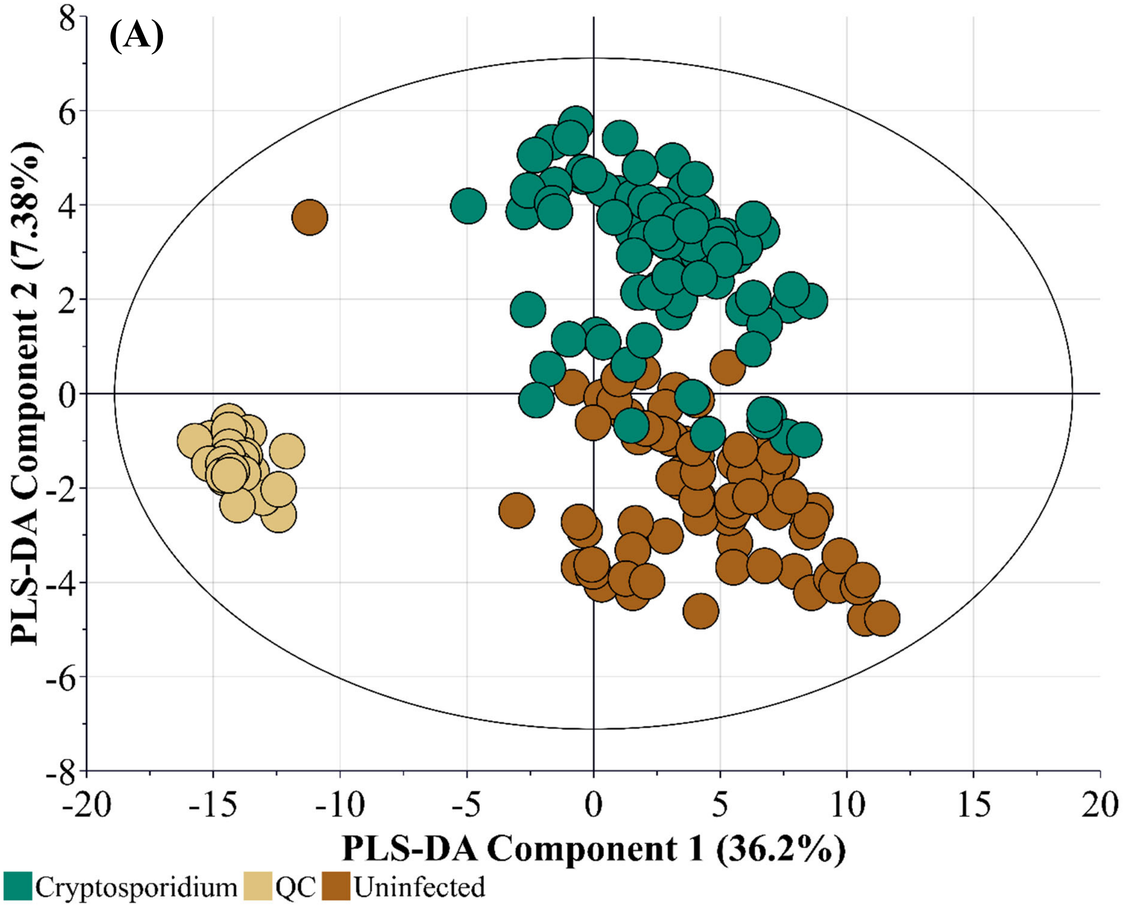

(A)
PLS-DA Component 2 (7.38%)
PLS-DA Component 1 (36.2%)
8
6
4
2
0
-2
-4
-6
-8
-20
-15
-10
-5
0
5
10
15
20
Cryptosporidium
QC
Uninfected

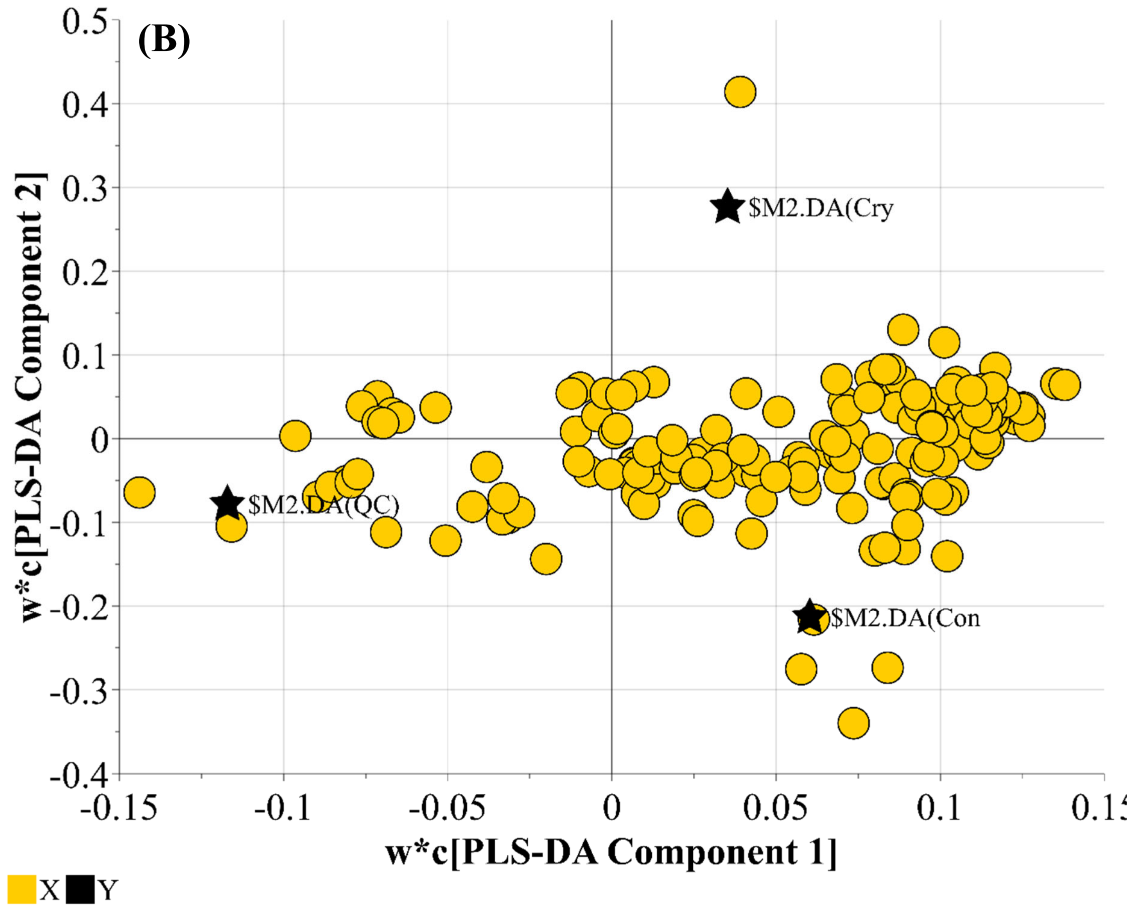

Figure S3. PLSDA plots of metabolic profile of various regions of intestine during cryptosporidiosis with respect to uninfected mice. The plots represent (A) PLS-DA Loading scatter for all samples (Uninfected, *C. parvum* infected and, QC = Quality Control). R^2X (cum) = 0.736, R^2Y (cum) = 0.981, Q^2 (cum) = 0.888.

Table S3. Major upregulated (FC ≥ 2) and downregulated (FC ≤ 0.5) metabolites in mouse duodenum during cryptosporidiosis with respect to the uninfected mice. ***Note:*** *The represented values were adjusted for Benjamini-Hochberg false discovery rate (FDR) in Metaboanalyst 4.0 FDR adjustment tool.*

**Metabolite**	**Fold Change**	**P value (adjusted)**
Citric acid	0.2359	0.0516
Trans-4-hydroxy-L-proline	0.1586	0.0611
Gluconic acid lactone	0.1362	0.0135
Sedoheptulose	0.1147	0.0351
Glucoheptonic acid	0.0955	0.0401

Glycerol	0.0766	0.0461
Allantoin	0.0686	$7.59e^{-06}$
O-acetylsalicylic acid	0.0086	0.0007
6-hydroxy caproic acid	$8.81e^{-06}$	0.0001
Succinic acid	$4.70e^{-07}$	0.0467

Table S4. Major upregulated (FC ≥ 2) and downregulated (FC ≤ 0.5) metabolites in mouse jejunum during cryptosporidiosis with respect to the uninfected mice. ***Note:*** *The represented values were adjusted for Benjamini-Hochberg false discovery rate (FDR) in Metaboanalyst 4.0 FDR adjustment tool.*

**Metabolite**	**Fold Change**	**P value (adjusted)**
Glycolic acid	0.4544	0.0131
L-mimosine	0.3225	0.0349
Oxalic acid	0.2635	0.0094
Gluconic acid lactone	0.2613	0.6894
α-D-Glucose	0.1657	0.0494
Maleic acid	0.1517	0.0078
Succinic acid	0.1378	0.0368
Allantoin	0.0930	0.0138
Glycerol	0.0765	0.0439
O-acetylsalicylic acid	0.0442	0.0006
Adenosine-5-monophosphate	0.0005	0.05078
6-hydroxy caproic acid	$1.36e^{-05}$	$4.86e^{-05}$

Table S5. Major upregulated (FC ≥ 2) and downregulated (FC ≤ 0.5) metabolites in mouse ileum during cryptosporidiosis with respect to the uninfected mice. ***Note:*** *The represented values were adjusted for Benjamini-Hochberg false discovery rate (FDR) in Metaboanalyst 4.0 FDR adjustment tool.*

**Metabolite**	**Fold Change**	**P value (adjusted)**
Compound_116	38.8510	0.0002
Shikimic acid	32.6580	0.0001
Gluconic acid lactone	0.0552	0.0002
Glycerol	0.0410	0.0004
Succinic acid	0.0402	0.0317
Ribitol	0.0222	0.0010
Allantoin	0.0133	0.0009
O-acetylsalicylic acid	0.0033	0.0001
Adenosine-5-monophosphate	0.0020	0.0314
4-hydroxypyridine	0.0006	0.0122
D-erythrose-4-phosphate	0.0004	0.0286
Tagatose	0.0001	0.0002
L- sorbose	0.0001	0.0005
Glucoheptonic acid	0.0001	0.0007
α-D-Glucose	0.0001	0.0008
Sedoheptulose	$4.74e^{-05}$	0.0014
6-hydroxy caproic acid	$2.91e^{-05}$	0.0002

Table S6. Major upregulated (FC ≥ 2) and downregulated (FC ≤ 0.5) metabolites in mouse caecum during cryptosporidiosis with respect to the uninfected mice. ***Note:*** *The represented values were adjusted for Benjamini-Hochberg false discovery rate (FDR) in Metaboanalyst 4.0 FDR adjustment tool.*

**Metabolite**	**Fold Change**	**P value (adjusted)**
Cellobiose	26982	$3.51e^{-05}$
Cycloleucine	26112	0.0006
L-glutamic acid	26107	0.0006
D-malic acid	7157.8	$3.67E^{-05}$
Sophorose	3645.1	0.0039
Compound_116	997	0.0001
Shikimic acid	8.4689	0.0045
D-sphingosine	5.4793	0.0240
L- lactic acid	2.7151	0.0186
α ketoglutaric acid	0.3294	0.0250
4-hydroxypyridine	0.2818	0.0444
L-lysine	0.2672	0.0310
Compound_22	0.1940	0.0100
L-tyrosine	0.0847	0.0002
Tagatose	0.0834	0.0002
Allantoin	0.0646	0.0001
Compound_86	0.0048	0.0452
O-acetylsalicylic acid	0.0013	$2.55e^{-06}$
Creatinine	0.0012	0.0001
Inosine 5'-monophosphate	$9.11e^{-05}$	0.0444
Adenosine-5-monophosphate	$9.11e^{-05}$	0.0444
6-hydroxy caproic acid	$1.00e^{-05}$	$2.57e^{-06}$
Succinic acid	$2.56e^{-06}$	0.0014

Table S7. Major upregulated (FC ≥ 2) and downregulated (FC ≤ 0.5) metabolites in mouse colon during cryptosporidiosis with respect to the uninfected mice. ***Note:*** *The represented values were adjusted for Benjamini-Hochberg false discovery rate (FDR).*

**Metabolite**	**Fold Change**	**P value (adjusted)**
Cellobiose	7152.5	$2.14e^{-08}$
Compound_127	4169.9	$2.03e^{-08}$
D-malic acid	2154.4	0.0005
Compound_122	3.9308	0.0049
3,4-dihydroxymandelic acid	3.8245	0.0578
Compound_93	2.0142	0.0021
Trans-aconitic acid	0.3555	0.0887
Trans-4-hydroxy-L-proline	0.2142	0.0549
Creatinine	0.1640	0.0091
L-lysine	0.1591	0.0021
Allantoin	0.1449	0.0636
D-mannose	0.1167	0.0008
Galactinol	0.1087	0.0024
Gluconic acid lactone	0.1006	0.0005
O-acetylsalicylic acid	0.0255	$1.52e^{-06}$
Cholic acid	0.0244	0.0035
Allantoin	0.0225	$1.08e^{-07}$
Glycine	0.0206	0.0271
L-methionine	0.0015	$4.04e^{-05}$
L-tyrosine	0.0005	0.0007
Glucoheptonic acid	$7.97e^{-05}$	$1.73e^{-08}$
α-D-Glucose	$6.43e^{-05}$	$1.73e^{-08}$
Sedoheptulose	$5.57e^{-05}$	$2.53e^{-08}$
6-hydroxy caproic acid	$3.20e^{-05}$	$6.71e^{-08}$

Table S8. Major upregulated (FC ≥ 2) and downregulated (FC ≤ 0.5) metabolites in mouse faeces during cryptosporidiosis with respect to the uninfected mice. ***Note:*** *The represented values were adjusted for Benjamini-Hochberg false discovery rate (FDR) in Metaboanalyst 4.0 FDR adjustment tool.*

**Metabolite**	**Fold Change**	**P value (adjusted)**
Cellobiose	14994	0.0066
Cycloleucine	10128	0.0036
L-glutamic acid	10125	0.0036
Compound_127	7653	0.0094
D-sphingosine	6668.9	0.0599
D-malic acid	2787.8	0.0094
Compound_129	2535.7	0.0599
Fumaric acid	726.78	0.0134
L-threonine	105.98	0.0277
Shikimic acid	82.828	0.0066
Compound_121	18.134	0.0134
D-ala-D-ala	17.421	0.0322
Adenosine	9.458	0.0198
Tartaric acid	2.8516	0.0277
Sedoheptulose	0.1441	0.0293
α-Glucose	0.1378	0.0293
Tagatose	0.1118	0.0198
Galactinol	0.1064	0.0198
L-tyrosine	0.1033	0.0198
Glucoheptonic acid	0.1031	0.0198
Gluconic acid lactone	0.0764	0.0277
L-methionine	0.0746	0.0148
Allantoin	0.0465	0.0188
Ribitol	0.0178	0.0134
Compound_86	0.0121	0.0337
2-isopropylmalic acid	0.0076	0.0277
Glycine	0.0008	0.0134
Dehydroascorbic acid	0.0006	0.0706
Allantoin	0.0003	0.0124

Cholic acid	0.0002	0.0198
Compound_22	0.0001	0.0148
L- sorbose	$8.71e^{-05}$	0.0134
Methyl-β-D-galactopyranoside	$3.88e^{-05}$	0.0153
6-hydroxy caproic acid	$1.98e^{-05}$	0.0198

Table S9. Signature host-response proteins expressed in mouse gut during cryptosporidiosis (combined output of duodenum: jejunum: ileum: caecum: colon: faeces) with respect to Giardiasis and UPEC infections when compared to the uninfected mice. The term cFC refers to log10 normalised combined fold change of statistically significant (adjusted p-value ≤ 0.05) proteins expressed.

Uniprot ID	Description	Crypto. (cFC)	P-value (FDR adj.)	Giardia (cFC)	P-value (FDR adj.)	UPEC (cFC)	P-value (FDR adj.)
E9Q2D1	Actin, cytoplasmic 1 (Fragment	7.26	$1.31e^{-16}$			6.02	$3.94e^{-20}$
E9Q606	Actin, cytoplasmic 1 (Fragment)	6.17	$3.3e^{-09}$	1.07	0.0444	1.87	0.0037
P68134	Actin, alpha skeletal muscle	5.37	$5.06e^{-09}$	6.20	$9.65e^{-18}$		
G3UZ07	Actin, cytoplasmic 2 (Fragment)	5.24	$1.88e^{-07}$			1.56	0.0204
E9Q5F4	Actin, cytoplasmic 1 (Fragment)	4.39	$4.41e^{-08}$			-1.45	0.0183
P02535	Keratin, type I cytoskeletal	3.70	$9.17e^{-06}$			1.57	0.0001
A2A513	Keratin, type I cytoskeletal 10	3.70	$9.17e^{-06}$			1.57	0.0001
P48962	ADP/ATP translocase 1	3.59	$1.7e^{-10}$				
A0A0U1RNK9	Electron transfer flavoprotein subunit beta (Fragment)	2.63	0.0036				
Q9DCW4	Electron transfer flavoprotein subunit beta	2.49	0.0012				
P11352	Glutathione peroxidase 1	2.38	0.0001	0.98	0.0054	2.11	0.0001
Q06890	Clusterin	2.35	0.0002			1.00	0.0478
P63260	Actin, cytoplasmic 2	2.33	$7.47e^{-07}$	-1.19	$1.23e^{-06}$	0.80	0.0020
P60710	Actin, cytoplasmic 1	2.33	$7.47e^{-07}$	-1.19	$1.23e^{-06}$	0.80	0.0020
D3Z5E2	Heat shock cognate 71 kDa protein (Fragment)	2.32	0.0003	-1.22	0.0097		

A0A1D5RM20	Actin, alpha skeletal muscle (Fragment)	2.30	0.0118	4.95	$1.48e^{-12}$		
P43276	Histone H1.5	2.27	0.0006				
A0A140LJ61	Synemin	2.21	0.0084				
Q9ET01	Glycogen phosphorylase, liver form	2.08	0.0012	1.87	0.0010		
Q3UEJ6	Alpha-1,4 glucan phosphorylase	2.08	0.0012	1.87	0.0010		
P31786	Acyl-CoA-binding protein	2.08	0.0006			-1.29	0.0113
Q4VWZ5	Acyl-CoA-binding protein	2.08	0.0006			-1.29	0.0113
P09411	Phosphoglycerate kinase 1	2.07	0.0282				
E9Q1F2	Actin, cytoplasmic 1	2.06	$5.46e^{-06}$	-1.33	$9.79e^{-08}$	0.78	0.0016

Table S10. Major upregulated (FC ≥ 2) and downregulated (FC ≤ 0.5) metabolites in serum during cryptosporidiosis with respect to the uninfected mice. ***Note:*** *The represented values were adjusted for Benjamini-Hochberg false discovery rate (FDR) in MetaboAnalyst 4.0 FDR adjustment tool.*

**Metabolite**	**Fold Change**	**P value (adjusted)**
Compound_110	10.525	0.0446
Cellobiose	6.2187	0.0222
Trans-aconitic acid	0.1039	0.0023
Compound_87	0.088	0.0089
Palmitoleic acid	0.0726	0.0145
Compound_53	0.0694	0.0192
O-acetylsalicylic acid	0.0573	0.0141
Oleic acid	0.047	0.0145
Compound_86	0.0454	0.0143
Dehydroascorbic acid1	0.0324	0.0222
Myristic acid	0.0269	0.0089
L-tyrosine	0.0175	0.0089
Compound_32	0.002	0.0089
Glycerol	0.0017	0.0065
Glycine	0.0005	0.0145
Cholic acid	0.0001	0.0065
Compound_33	0.0001	0.0089

Table S11. Major upregulated (FC ≥ 2) and downregulated (FC ≤ 0.5) metabolites in liver during cryptosporidiosis with respect to the uninfected mice. ***Note:*** *The represented values were adjusted for Benjamini-Hochberg false discovery rate (FDR) in MetaboAnalyst 4.0 FDR adjustment tool.*

**Metabolite**	**Fold Change**	**P value (adjusted)**
L-mimosine	11521	0.0123
Oxalic acid	536.45	0.0406
L-fucose	3.9177	0.0056
Succinic acid	0.2898	0.0046
Compound_32	0.1401	0.0226
Glycerol	0.1336	0.0225
Compound_33	0.0877	0.0154
Compound_25	0.0766	0.0154
6-hydroxy caproic acid	0.0624	0.0157

Table S12. Signature host-response proteins expressed in mouse serum-liver during cryptosporidiosis with respect to the uninfected mice.

**UniProt ID**	**Description**	**Combined Log (FC)**	**P value (FDR adj.)**
E9Q223	Hemoglobin, β adult s chain (Fragment) OS=*Mus musculus* OX=10090 GN=Hbb-bs PE=1 SV=1	10.63	2.97E-11
Q9CQ52	Chymotrypsin-like elastase family member 3B OS=*Mus musculus* OX=10090 GN=Cela3b PE=1 SV=1	4.94	0.0001
A0A0A6YWP4	Complement factor H (Fragment) OS=*Mus musculus* OX=10090 GN=Cfh PE=1 SV=1	3.97	0.0008
A0A2K6EDJ7	Inter α-trypsin inhibitor, heavy chain 4 OS=*Mus musculus* OX=10090 GN=Itih4 PE=1 SV=1	3.91	0.0059
Q8VCU2	Glycosylphosphatidylinositol specific phospholipase D1 OS=*Mus musculus* OX=10090 GN=Gpld1 PE=1 SV=1	3.90	1.09E-05
O70362	Phosphatidylinositol-glycan-specific phospholipase D OS=*Mus musculus* OX=10090 GN=Gpld1 PE=1 SV=1	3.90	1.09E-05
A0A0A6YXS8	Antithrombin-III OS=*Mus musculus* OX=10090 GN=Serpinc1 PE=1 SV=1	3.70	0.0008
Q9DBD0	Inhibitor of carbonic anhydrase OS=*Mus musculus* OX=10090 GN=Ica PE=1 SV=1	3.67	0.0067
D3YY36	RIKEN cDNA 1300017J02 gene OS=*Mus musculus* OX=10090 GN=1300017J02Rik PE=1 SV=1	3.67	0.0067
E9Q5L2	Inter α-trypsin inhibitor, heavy chain 4 OS=*Mus musculus* OX=10090 GN=Itih4 PE=1 SV=1	3.65	0.0133

UniProt ID	Description	Combined Log (FC)	P value (FDR adj.)
E9Q8Y5	Clusterin (Fragment) OS=*Mus musculus* OX=10090 GN=Clu PE=1 SV=1	3.53	0.0002
H3BK95	Complement factor B (Fragment) OS=*Mus musculus* OX=10090 GN=Cfb PE=4 SV=1	3.51	0.0005
B0R0E9	Creatine kinase U-type, mitochondrial (Fragment) OS=*Mus musculus* OX=10090 GN=Ckmt1 PE=1 SV=1	3.27	0.0111
F6W2T4	Complement factor B (Fragment) OS=*Mus musculus* OX=10090 GN=Cfb PE=3 SV=1	3.21	0.0006
H3BLB8	Paraoxonase 1, isoform CRA_c OS=*Mus musculus* OX=10090 GN=Pon1 PE=1 SV=1	3.18	0.0199
G3X9T8	Ceruloplasmin OS=*Mus musculus* OX=10090 GN=Cp PE=1 SV=1	3.11	0.0102
E9Q9B8	Clusterin (Fragment) OS=*Mus musculus* OX=10090 GN=Clu PE=1 SV=1	3.03	0.0099
P08226	Apolipoprotein E OS=*Mus musculus* OX=10090 GN=Apoe PE=1 SV=2	3.03	0.0113
A2ARP5	Creatine kinase U-type, mitochondrial (Fragment) OS=*Mus musculus* OX=10090 GN=Ckmt1 PE=1 SV=1	2.99	0.0041
Q5ND35	A-2-antiplasmin (Fragment) OS=*Mus musculus* OX=10090 GN=Serpinf2 PE=1 SV=1	2.97	0.0043
Q06890	Clusterin OS=*Mus musculus* OX=10090 GN=Clu PE=1 SV=1	2.93	0.0011
A0A0N4SVU1	Predicted gene 7298 OS=*Mus musculus* OX=10090 GN=Gm7298 PE=4 SV=1	2.89	0.0040

UniProt ID	Description	Combined Log (FC)	P value (FDR adj.)
A0A1B0GX15	Apolipoprotein E (Fragment) OS=*Mus musculus* OX=10090 GN=Apoe PE=1 SV=1	2.89	0.0133
P01029	Complement C4-B OS=*Mus musculus* OX=10090 GN=C4b PE=1 SV=3	2.87	0.0181
G3X977	Inter-α trypsin inhibitor, heavy chain 2 OS=*Mus musculus* OX=10090 GN=Itih2 PE=1 SV=1	2.82	0.0127
P28665	Murinoglobulin-1 OS=*Mus musculus* OX=10090 GN=Mug1 PE=1 SV=3	2.82	0.0094
G3UZM8	Apolipoprotein E (Fragment) OS=*Mus musculus* OX=10090 GN=Apoe PE=1 SV=1	2.81	0.0254
P11680	Properdin OS=*Mus musculus* OX=10090 GN=Cfp PE=2 SV=2	2.76	0.0133
A0A075B5P6	Immunoglobulin heavy constant mu (Fragment) OS=*Mus musculus* OX=10090 GN=Ighm PE=1 SV=1	2.76	0.0119
D3YUI3	Pregnancy zone protein (Fragment) OS=*Mus musculus* OX=10090 GN=Pzp PE=1 SV=1	2.75	0.0268
H7BWY6	Retinol-binding protein 4 OS=*Mus musculus* OX=10090 GN=Rbp4 PE=1 SV=1	2.75	0.0185
A0A075B6A0	Immunoglobulin heavy constant mu (Fragment) OS=*Mus musculus* OX=10090 GN=Ighm PE=1 SV=2	2.74	0.0120
D3Z2B2	Kininogen-1 (Fragment) OS=*Mus musculus* OX=10090 GN=Kng1 PE=1 SV=8	2.72	0.0128
A0A1B0GS57	Hemopexin (Fragment) OS=*Mus musculus* OX=10090 GN=Hpx PE=1 SV=1	2.72	0.0084

**UniProt ID**	**Description**	**Combined Log (FC)**	**P value (FDR adj.)**
P30275	Creatine kinase U-type, mitochondrial OS=*Mus musculus* OX=10090 GN=Ckmt1 PE=1 SV=1	2.71	0.0053
A0A0G2JGM6	Vitamin D-binding protein (Fragment) OS=*Mus musculus* OX=10090 GN=Gc PE=1 SV=1	2.71	0.0106
P01872	Immunoglobulin heavy constant mu OS=*Mus musculus* OX=10090 GN=Ighm PE=1 SV=2	2.69	0.0133
E9QP56	Apolipoprotein C-III OS=*Mus musculus* OX=10090 GN=Apoc3 PE=1 SV=1	2.63	0.0094
Q61838	Pregnancy zone protein OS=*Mus musculus* OX=10090 GN=Pzp PE=1 SV=3	2.57	0.0253
P09813	Apolipoprotein A-II OS=*Mus musculus* OX=10090 GN=Apoa2 PE=1 SV=2	2.57	0.0013
A0A0R4J0I1	MCG1051009 OS=*Mus musculus* OX=10090 GN=Serpina3k PE=1 SV=1	2.55	0.0161
P01865	Ig γ-2A chain C region, membrane-bound form OS=*Mus musculus* OX=10090 GN=Igh-1a PE=1 SV=3	2.54	0.0297
E9PYP1	Carboxylic ester hydrolase OS=*Mus musculus* OX=10090 GN=Ces1a PE=1 SV=1	2.52	0.0341
Q80X76	Serine protease inhibitor A3F OS=*Mus musculus* OX=10090 GN=Serpina3f PE=1 SV=3	2.47	0.0333
A0A0A6YXW6	Immunoglobulin heavy constant α (Fragment) OS=*Mus musculus* OX=10090 GN=Igha PE=1 SV=1	2.44	0.0054
P07309	Transthyretin OS=*Mus musculus* OX=10090 GN=Ttr PE=1 SV=1	2.40	0.0121

UniProt ID	Description	Combined Log (FC)	P value (FDR adj.)
E9Q499	Serine protease inhibitor A3G (Fragment) OS=*Mus musculus* OX=10090 GN=Serpina3g PE=1 SV=8	2.36	0.0436
Q61247	A-2-antiplasmin OS=*Mus musculus* OX=10090 GN=Serpinf2 PE=1 SV=1	2.34	0.0119
P52430	Serum paraoxonase/arylesterase 1 OS=*Mus musculus* OX=10090 GN=Pon1 PE=1 SV=2	2.31	0.0492
P01027	Complement C3 OS=*Mus musculus* OX=10090 GN=C3 PE=1 SV=3	2.30	0.0374
Q01339	B-2-glycoprotein 1 OS=*Mus musculus* OX=10090 GN=Apoh PE=1 SV=1	2.25	0.0203
H9H9R5	Plasma kallikrein OS=*Mus musculus* OX=10090 GN=Klkb1 PE=1 SV=2	2.24	0.0366
Q91X72	Hemopexin OS=*Mus musculus* OX=10090 GN=Hpx PE=1 SV=2	2.21	0.0325
Q07456	Protein AMBP OS=*Mus musculus* OX=10090 GN=Ambp PE=1 SV=2	2.20	0.0407
A0A0A6YX70	Antithrombin-III (Fragment) OS=*Mus musculus* OX=10090 GN=Serpinc1 PE=1 SV=1	2.18	0.0251
Q8K0C5	Zymogen granule membrane protein 16 OS=*Mus musculus* OX=10090 GN=Zg16 PE=1 SV=1	2.16	0.0043
P21614	Vitamin D-binding protein OS=*Mus musculus* OX=10090 GN=Gc PE=1 SV=2	2.15	0.0186
F7CJN9	Transferrin (Fragment) OS=*Mus musculus* OX=10090 GN=Trf PE=4 SV=1	2.15	0.0360

**UniProt ID**	**Description**	**Combined Log (FC)**	**P value (FDR adj.)**
I7HPW5	B-2-glycoprotein 1 (Fragment) OS=*Mus musculus* OX=10090 GN=Apoh PE=1 SV=1	2.14	0.0116
I7HJR3	B-2-glycoprotein 1 (Fragment) OS=*Mus musculus* OX=10090 GN=Apoh PE=1 SV=1	2.12	0.0282
Q00897	A-1-antitrypsin 1-4 OS=*Mus musculus* OX=10090 GN=Serpina1d PE=1 SV=1	2.11	0.0355
F7BAE9	Transferrin (Fragment) OS=*Mus musculus* OX=10090 GN=Trf PE=1 SV=1	2.10	0.0366
E9Q035	Predicted gene 20425 OS=*Mus musculus* OX=10090 GN=Gm20425 PE=4 SV=1	2.06	0.0413
Q921I1	Serotransferrin OS=*Mus musculus* OX=10090 GN=Tf PE=1 SV=1	2.04	0.0418
P01837	Immunoglobulin kappa constant OS=*Mus musculus* OX=10090 GN=Igkc PE=1 SV=2	2.04	0.0071
D6RGQ0	Complement factor H OS=*Mus musculus* OX=10090 GN=Cfh PE=1 SV=1	2.02	0.0206

Table S13. Variable Importance in Projection (VIP) scores and impact importance of metabolites in small intestine (A: Jejunum, B: ileum) during cryptosporidiosis with respect to the uninfected mice. VIP score ≥ 2 were considered significantly impacting the overall metabolic behaviour.

**Metabolite**	**Duodenum**	**Jejunum**	**Ileum**	**Caecum**	**Colon**	**Faeces**
Glycerol	5.77	5.93	6.06	4.79	5.81	2.78
Succinate	5.76	1.05	5.58	4.66	2.49	1.36
Citrate	5.45	1.15	0.63	5.53	6.31	0.97
Orthophosphate	5.25	6.62	3.73	5.58	7.67	0.06
Glycolate	3.13	3.98	2.24	2.26	3.73	0.00
Glucose	2.28	2.34	0.83	2.27	1.72	0.64
Sedoheptulose	2.04	0.89	0.52	1.83	0.40	0.41
Methyl-beta-D-galactose	1.79	0.15	0.56	0.27	0.43	0.43
Cellobiose	1.53	3.70	5.85	0.48	0.26	0.48
Oxalate	1.53	1.66	2.73	3.03	1.87	4.88
Malonate	1.41	1.30	2.25	2.66	0.94	4.13
Lactate	1.26	0.43	0.91	1.41	0.58	0.00
Melezitose	1.26	0.97	0.63	0.48	0.03	0.00
Shikimate	1.23	0.49	0.18	0.26	0.28	0.23
Glucoheptonate	1.19	0.54	0.36	0.98	0.28	0.30
Glutamate	0.79	0.30	0.49	2.68	0.48	4.53
Urea	0.68	0.91	0.71	1.00	0.80	1.97

Metabolite	Duodenum	Jejunum	Ileum	Caecum	Colon	Faeces
Sucrose	0.64	1.19	0.38	0.04	0.04	0.11
6-hydroxy caproate	0.63	0.53	0.84	1.16	0.70	0.80
L-norleucine	0.60	0.56	1.01	0.04	0.00	0.02
2-isopropylmalate	0.57	0.05	0.10	0.31	0.32	0.16
Tagatose	0.56	0.12	0.20	0.27	0.09	0.17
Sorbose	0.54	0.11	0.20	0.23	0.11	0.20
Sphingosine	0.54	1.17	1.91	0.13	0.03	0.15
Gluconate lactone	0.49	0.23	0.24	0.28	0.22	0.18

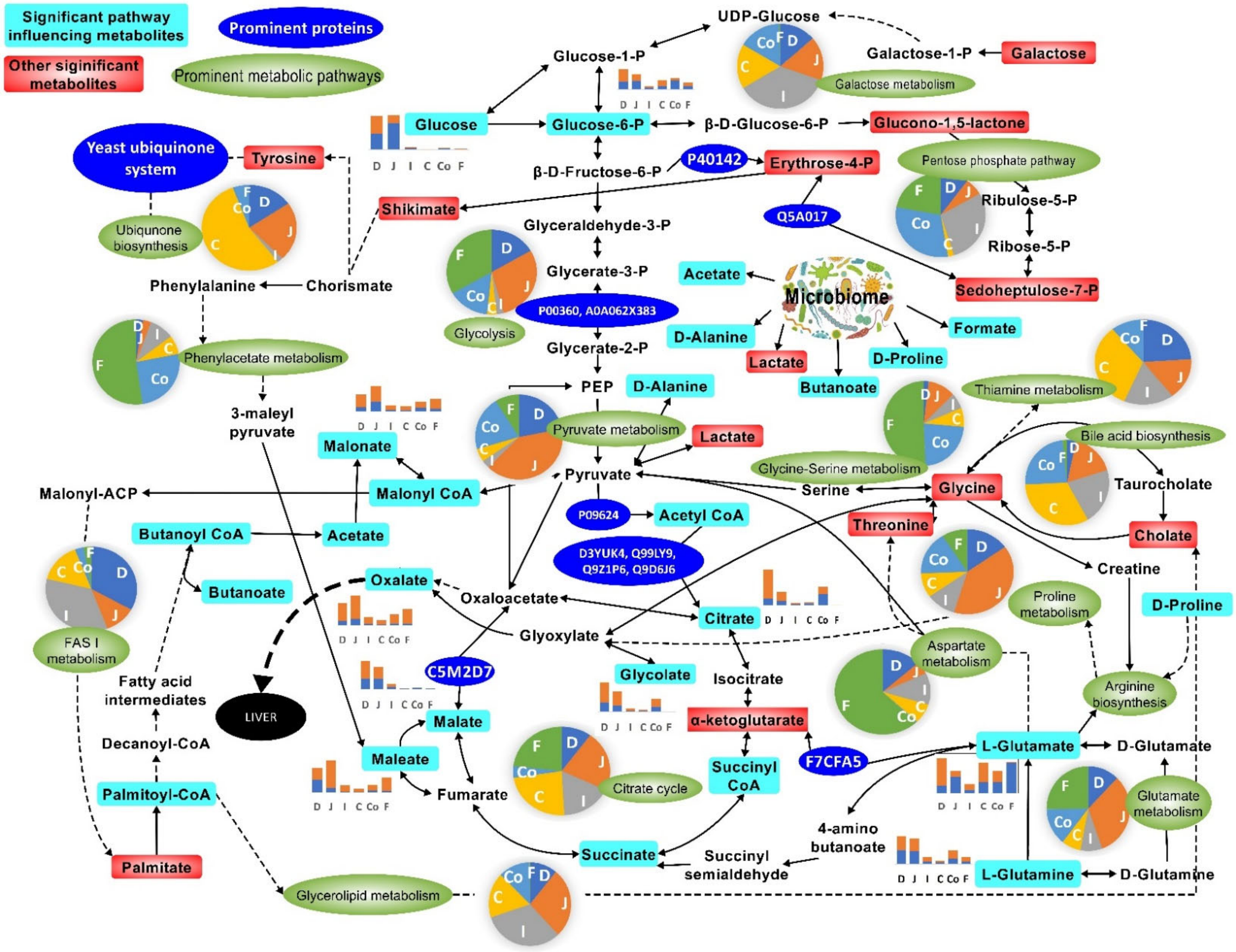

Figure S8. Predominant metabolic pathways in mouse gastrointestinal tract during cryptosporidiosis.
